# PSMD11 stabilizes PGM3 by antagonizing Parkin to promote bladder cancer progression through energy metabolism reprogramming

**DOI:** 10.1038/s41419-026-08691-4

**Published:** 2026-04-06

**Authors:** Yu Cheng, Tao Chen, Guanghao Zheng, Zhen Song, Gan Zhang, Song Xiao, Xuepeng Rao, Tao Zeng

**Affiliations:** Department of Urology, The Second Affiliated Hospital, Jiangxi Medical College, Nanchang University, Nanchang, Jiangxi China

**Keywords:** Bladder cancer, Cancer metabolism

## Abstract

Bladder cancer (BCa), a highly prevalent and aggressive tumor of the urinary system, typically exhibits poor clinical outcomes, particularly in advanced stages where therapeutic efficacy remains inadequate. A key characteristic of tumorigenesis, metabolic reprogramming, contributes substantially to cancer cell proliferation and metastatic progression. In the current investigation, phosphoglucomutase 3 (PGM3) was markedly overexpressed in BCa tissues, with elevated PGM3 expression strongly associated with unfavorable prognosis. Downregulation of PGM3 inhibited BCa tumor growth and metastasis by suppressing energy metabolism pathways, including glycolysis and oxidative phosphorylation (OXPHOS). Mechanistically, proteasome 26S subunit non-ATPase 11 (PSMD11) interacted with PGM3, reducing its ubiquitination and proteasomal degradation. Additionally, Parkin acted as a ubiquitinase, destabilizing PGM3, whereas PSMD11 competed with Parkin for PGM3 binding, thereby attenuating Parkin-mediated ubiquitination and stabilizing PGM3. Further analysis demonstrated that PSMD11 enhanced glycolysis and OXPHOS through PGM3, promoting BCa malignancy. Higher PSMD11 expression positively correlated with increased PGM3 expression. Collectively, these findings suggest that targeting the PSMD11/PGM3 axis could provide a promising therapeutic strategy for BCa.

## Introduction

Globally, bladder cancer (BCa) represents a significant urological malignancy and ranks as the ninth most common cancer worldwide [1]. In 2022, an estimated 613,791 new diagnoses and approximately 220,349 related deaths were reported globally [1]. Urothelial carcinoma remains the most common histological form, comprising roughly 75% of cases classified as non-muscle-invasive BCa (NMIBC), with the remainder categorized as muscle-invasive BCa (MIBC) [2]. The progression risk from NMIBC to MIBC varies based on tumor grade and invasion depth, with around 20% of NMIBC patients advancing to MIBC within two years post-diagnosis [3]. Despite aggressive surgical interventions, such as radical cystectomy combined with pelvic lymphadenectomy, metastasis still develops in approximately half of MIBC patients, and the overall five-year survival rate does not exceed 30% [4]. Hence, exploring the molecular mechanisms responsible for BCa progression and discovering novel therapeutic strategies is essential.

Metabolic reprogramming is a key feature of malignant transformation, significantly contributing to tumor growth and metastasis [5, 6]. Cancer cells exhibit a distinct bioenergetic profile characterized by a preference for aerobic glycolysis over mitochondrial oxidative phosphorylation (OXPHOS). This metabolic adaptation, termed the “Warburg effect,” allows rapid proliferation in hypoxic and nutrient-limited microenvironments [7, 8]. Such altered metabolic phenotypes have attracted attention as potential therapeutic vulnerabilities in cancer treatment. Current pharmacological strategies involve developing selective inhibitors targeting key glycolytic enzymes and kinases. These inhibitors aim to disrupt enhanced glycolytic activity, restore mitochondrial respiration initially, and ultimately induce apoptosis in cancer cells [9–11]. However, therapeutic efficacy is often limited by metabolic plasticity, as many tumor cells retain functional mitochondrial networks. This adaptability allows cancer cells to evade glycolytic inhibition by shifting energy production to OXPHOS-derived ATP generation, thereby overcoming metabolic stress and ensuring survival [12–15]. Thus, simultaneously targeting aerobic glycolysis and OXPHOS may represent a more effective therapeutic approach.

Phosphoglucomutase 3 (PGM3), known alternatively as N-acetylglucosamine-phosphate mutase 1 (AGM1), is an essential enzyme within the hexosamine biosynthetic pathway (HBP). This enzyme facilitates the conversion between GlcNAc-1-phosphate and GlcNAc-6-phosphate, ultimately synthesizing UDP-N-acetylglucosamine (UDP-GlcNAc). UDP-GlcNAc is an indispensable nucleotide sugar, critically required for glycosylation reactions, including N-linked and O-linked protein glycosylation [16]. Moreover, PGM3, belonging to the phosphoglucomutase enzyme group, governs the bidirectional conversion between glucose-6-phosphate (G-6-P) and glucose-1-phosphate (G-1-P). Specifically, G-1-P is transformed into G-6-P during glycogen breakdown, subsequently entering glycolysis. Conversely, conversion from G-6-P back to G-1-P yields UDP-glucose, an essential precursor for synthesizing various biomolecules, particularly glycoconjugates [17]. Recent studies revealed that inhibition of PGM3 effectively suppresses tumor progression and proliferation across diverse malignancies [18–20], and glioblastoma [21]. Notably, substantial antitumor activity was documented for the selective PGM3 inhibitor FR054 in these studies [18–20]. Despite these findings, the exact role and regulatory mechanisms of PGM3 in BCa have not yet been fully clarified. Additionally, previous research predominantly emphasized PGM3’s influence on tumor progression through its regulation of the HBP pathway. It remains unknown whether PGM3 additionally affects other glucose metabolic pathways.

The present study identified increased PGM3 expression within BCa samples, and elevated PGM3 levels strongly correlated with unfavorable patient outcomes. Silencing PGM3 expression significantly reduced BCa tumor growth and metastasis, mainly by restraining glycolysis and OXPHOS. Mechanistically, proteasome 26S subunit non-ATPase 11 (PSMD11) interacted with PGM3, reducing its ubiquitination and proteasomal degradation. Further analyses demonstrated that Parkin acted as a ubiquitinase, destabilizing PGM3. In contrast, PSMD11 attenuated Parkin-mediated ubiquitination by competing with Parkin for binding to PGM3, resulting in PGM3 stabilization. Higher PSMD11 expression positively correlated with increased PGM3 levels. Therefore, targeting the PSMD11/PGM3 axis may provide a promising therapeutic strategy for BCa.

## Materials and Methods

### Clinical samples

In this research, paired tissue samples (*n* = 39) from bladder carcinoma and corresponding adjacent non-tumorous regions were collected at the Second Affiliated Hospital of Nanchang University. All specimens were obtained following rigorous ethical standards and guidelines, and each participant provided written informed consent prior to involvement. Ethical approval for this study was granted by the Medical Ethics Committee of the Second Affiliated Hospital of Nanchang University.

### Immunohistochemistry (IHC)

Initially, tissue sections underwent deparaffinization, followed by a 10-minute treatment with 3% hydrogen peroxide solution to eliminate endogenous peroxidase activity. Antigen retrieval was conducted using an autoclave heating method in citrate buffer for 30 minutes. Subsequently, sections were rinsed using PBS and incubated at 4°C overnight with selected primary antibodies. Following incubation, HRP-linked secondary antibodies were applied for 1 hour. Visualization was achieved using diaminobenzidine, and slides were counterstained with hematoxylin. Two independent pathologists performed slide evaluation employing the IRS scoring system. Detailed information on the antibodies used in the study is shown in Supplementary Table S1.

### Western blot (WB)

Cellular proteins were extracted in RIPA lysis buffer (Beyotime, China) containing the protease inhibitor PMSF. Protein quantification was determined by employing a BCA protein assay kit (TransGen Biotech, China). Protein samples were heat-denatured at 100°C for 5–10 minutes in loading buffer prior to electrophoresis. SDS-PAGE separated proteins were transferred onto PVDF membranes, which were then incubated in blocking buffer (5% milk) for 2 hours to minimize non-specific binding. After washing thrice with TBST, membranes underwent overnight incubation at 4°C with specific primary antibodies (Proteintech, China). The following day, secondary antibody (Proteintech, China) incubation occurred for 1 hour. Protein detection was subsequently performed using enhanced chemiluminescence visualization after three additional TBST washes. Detailed information of the antibodies used in the study is shown in Supplementary Table S1.

### Cell lines and cell culture

Cell lines HEK293T, T24, and 5637 were commercially sourced from ATCC. DMEM (Gibco, USA) was utilized for cultivating HEK293T and T24 cells, whereas RPMI-1640 medium (Gibco, USA) was used to culture 5637 cells. Each medium was supplemented with 10% fetal bovine serum (FBS) and 1% penicillin-streptomycin. Regular screening for mycoplasma contamination was conducted using real-time quantitative PCR (RT-qPCR). All cells were cultured at 37°C with 5% CO_2_.

### Cell transfection

Short hairpin RNAs (shRNAs) (Supplementary Table S2) specific for targeting PGM3 and PSMD11 were synthesized by Obio Biotechnology (Shanghai, China). Following lentiviral-mediated gene transfer, stable cell populations were selected using puromycin at a concentration of 2 ug/ml. Flag-PGM3 plasmids, HA-PSMD11 plasmids, Myc-Ub plasmids (WT, K6, K11, K27, K29, K33, K48, K63, K6R, K11R, K27R, K29R, K33R, K48R, K63R), site-mutant plasmids of PGM3 (K15R, K186R, K189R, K350A, H358A, R460A, T465A, R460A & T465A), PGM3 domain-deletion mutant plasmids (PGM3ΔA, deletion active serine domain; PGM3ΔM, deletion Mg2+ binding domain; PGM3ΔS, deletion sugar binding domain; PGM3ΔP, deletion phosphate binding domain), His-Parkin plasmids and His-Parkin-C431S mutant plasmids as well as each control plasmid were purchased from MiaoLing Biology (Wuhan, China). Lipofectamine™ 3000 reagent (Invitrogen, USA) was applied to achieve transient gene transfer. Transfection efficiency was verified by RT-qPCR and WB assays.

### RT-qPCR

Cellular RNA extraction was carried out with Trizol reagent supplied by Tiangen Biotech (China). cDNA was subsequently produced using a high-efficiency reverse transcription kit (Tiangen Biotech, China). Synthesized cDNA samples served as templates for quantitative PCR amplification, executed on the StepOnePlus™ Real-time PCR System. Primer sequences employed for RT-qPCR are provided in Supplementary Table S3.

### Cell Counting Kit-8 (CCK-8) assay

To evaluate cellular proliferation, 2000 cells were introduced into individual wells of 96-well microplates, followed by incubation in standard culture conditions (37°C, 5% CO_2_) at defined time points: 24, 48, 72, 96, 120 hours. Subsequently, each well received 10 μL of CCK-8 reagent (APE BIO, USA), incubated further for 1 hour. Absorbance measurements at a wavelength of 450 nm were recorded utilizing a microplate reader.

### Colony formation assay

Cells (500-1000 cells/well) were seeded into 6-well plates and cultured for 7–10 days, replacing medium every 72 hours. Following incubation, the growth medium was aspirated, and cells underwent three PBS rinses. Cells were fixed with 4% paraformaldehyde for 30 minutes, stained for 15 minutes using 1% crystal violet solution, and colonies were then counted by processing digital images with ImageJ software.

### Transwell migration and invasion assays

BCa cells suspended in serum-free medium were placed into upper chambers of transwell devices, while lower chambers were filled with medium supplemented with 10% FBS. After incubation intervals of either 24 or 48 hours, cells traversing the membrane were fixed in 4% paraformaldehyde solution for 30 minutes, stained with crystal violet solution for 15 minutes, photographed with an inverted microscope, and finally quantified using ImageJ software.

### Wound healing assay

BCa cells were seeded into 6-well culture plates and grown to achieve nearly complete monolayer confluency in media supplemented with 10% FBS. A sterile pipette tip (1000 uL) was then employed to generate a uniform scratch across the monolayer surface. After rinsing twice with PBS solution, cells were maintained in serum-free conditions to eliminate proliferative effects. Images of scratch gaps were taken immediately after wounding (0 h) and at 24 h under an inverted microscopy system. The rate of cell migration was assessed by analyzing changes in scratch closure area using ImageJ software.

### Metabolite detection and analysis

Cells harvested from culture plates were promptly resuspended in pre-chilled 80% methanol, followed by vigorous mixing via vortex. The suspensions were incubated on ice for 5 min and centrifuged at 15,000 *g* (4 °C) for 15 min. Subsequently, 180 uL aliquots of clarified supernatants were transferred through protein precipitation filters prior to quantification and identification using liquid chromatography-tandem mass spectrometry (LC-MS/MS). Targeted metabolite profiling was executed using a Waters ACQUITY H-ClassD UPLC connected to a QTRAP 6500+ mass spectrometry instrument. Collected raw LC-MS/MS data were analyzed with Analyst software (v1.6.3).

### RNA sequencing (RNA-seq) analysis

OE Biotech (Shanghai, China) conducted RNA sequencing experiments. Cell-derived RNA was isolated using TRIzol reagent (Invitrogen, CA, USA). RNA purity and concentration were determined through spectrophotometric measurement on a NanoDrop 2000 device (Thermo Scientific, USA). Integrity of extracted RNA samples was assessed via Agilent 2100 Bioanalyzer (Agilent Technologies, USA). VAHTS Universal V6 RNA-seq Library Prep Kit was employed for library preparation, followed by sequencing performed on an Illumina NovaSeq 6000 sequencing platform.

### Oxygen consumption rate (OCR) and extracellular acidification rate (ECAR) analysis

Cells seeded in XF96 plates at a density of approximately 1.0 × 10^4^ cells per well were incubated overnight. After replacement with XF assay medium and further incubation for 1 h, OCR measurements were conducted utilizing a Cell Mito Stress Test Kit (Agilent, 103015-100). OCR was evaluated following successive additions of oligomycin (1 μM), FCCP (0.5 μM), and rotenone (1 μM). Likewise, ECAR was assessed via the Glycolysis Stress Test Kit (Agilent, 103020-100), involving sequential administration of glucose (10 mM), oligomycin (1 μM), and 2-deoxy-D-glucose (2-DG, 100 mM). Both OCR and ECAR data acquisition were performed using a Seahorse XF96 Analyzer (Seahorse Bioscience), and readings were normalized relative to cell counts in individual wells.

### Measurement of glucose uptake, ATP production, lactate levels, and NAD + /NADH ratio

For metabolite measurements, cells (1.0 × 10^6^) were seeded into six-well culture dishes and allowed to attach for approximately 8 h. The original medium was subsequently replaced with serum-free medium for an additional 24 h. Cell culture supernatants were harvested, and extracellular glucose and lactate concentrations were measured using commercially obtained kits (Glucose Assay Kit, Beyotime S0201S; Lactate Assay Kit, Beyotime S0208S). For ATP production and NAD⁺/NADH assessments, cells were lysed, followed by centrifugation (12,000 *g*, 5 min, 4 °C). The supernatants were then collected and analyzed with ATP assay kit (Beyotime, S0026) and NAD⁺/NADH assay kit (Beyotime, S0175) to determine ATP content and NAD^+^/NADH ratios, respectively.

### Mass spectrometry (MS) analysis

Flag-tagged PGM3-expressing plasmids were transfected into T24 cells. Anti-Flag magnetic beads (YEASEN, 20565ES03) were subsequently utilized to immunoprecipitated target proteins from cell lysates by incubating overnight at 4°C. After immunoprecipitation, complexes attached to the beads underwent thorough washing five times with RIPA buffer containing both phosphatase and protease inhibitors. The captured proteins were eluted and subsequently subjected to either silver staining or immunoblotting assays. For protein identification via LC-MS/MS, immunoprecipitated protein complexes obtained in parallel experiments were sequentially eluted thrice using lysis buffer, digested enzymatically with trypsin directly in solution, and analyzed by LC-MS/MS.

### Co-immunoprecipitation (Co-IP)

Cell extracts were prepared by lysing cells on ice for 30 minutes using RIPA buffer (Beyotime, China). The lysates were then centrifuged at 4 °C, after which the resulting supernatants were collected and incubated with designated primary antibodies at 4 °C overnight. Afterwards, recombinant Protein G magnetic beads (YEASEN, 36419ES03) were added, and samples were rotated gently overnight at 4 °C. Following five cycles of bead washing with buffer, the bound protein complexes were released by boiling in SDS-PAGE loading buffer for 10 minutes at 100 °C. Proteins recovered via Co-IP were separated electrophoretically on SDS-polyacrylamide gels and subsequently detected by WB analysis.

### Immunofluorescence (IF)

T24 and 5637 cells were seeded uniformly on sterile glass coverslips and cultured under standard conditions for predetermined intervals. Cells were fixed at ambient temperature for 30 minutes using 4% paraformaldehyde. Subsequently, primary antibody incubation was performed overnight at 4 °C. The following day, cells underwent PBS washes three times, and fluorescently labeled secondary antibodies were added, incubating for 1 hour. After another three washes with PBS, nuclei were labeled by counterstaining with DAPI dye for 20 minutes. Fluorescence images were then acquired using a confocal laser scanning microscope.

### Animal models

Male BALB/c nude mice, aged between 4 and 6 weeks, were procured from Hangzhou Ziyuan Laboratory Animal Technology. All animal procedures conformed strictly to the ethical standards outlined by the Institutional Animal Care and Use Committee (IACUC) of Nanchang Royo Biotech Co., Ltd. (approval ID: RYE2025031003). For establishing xenografts, T24 cells (5 × 10^6^ cells) were suspended in a combined solution (1:1 PBS:Matrigel) totaling 200 μL and injected subcutaneously into the axillary region of mice. Starting at day 6 after inoculation, tumor dimensions were recorded every third day using digital calipers. When indicated, drug administration was initiated at day 6 through daily intraperitoneal injections. Once tumors reached the preset endpoint volumes, animals were humanely euthanized, and tumor tissues were excised, weighed, photographed, and further processed for immunohistochemical analysis. Growth curves reflecting tumor progression were accordingly generated. For the lung metastasis model, the indicated shNC, shPGM3, shPSMD11, or shPSMD11+OE-PGM3 T24 cells (1 × 10^6^) transduced with luciferase were injected into the mice via the tail vein (3 mice per group) to model tumor metastasis. Lung metastasis detection began on the 20th day post-injection. Mice received an injection of 5 mg/ kg D-Luciferin (YEASEN, 40902ES02) and were imaged after 5 min using a LAGO imager with Aura imaging software (Spectral Instruments Imaging). Two weeks later, the mice were euthanized. Experienced researchers then quantified the number of pulmonary metastases through visual assessment.

### Statistical analysis

Data were reported as mean ± standard deviation (SD). GraphPad Prism (version 9.0) was used for performing statistical analyses and plotting figures. Unpaired Student’s *t*-test was used for comparisons between two unpaired groups, while paired Student’s *t*-test was used for comparisons between two paired groups. Statistical significance was defined as *P* < 0.05, where (*) represents *P* < 0.05, (**) represents *P* < 0.01, (***) represents *P* < 0.001.

## Results

### PGM3 is identified as a downstream gene of RAB14 and closely associated with BCa

Our research team has extensively explored the molecular events driving the initiation and progression of BCa [22–24]. Previously, we found that RAB14 promoted BCa tumorigenesis through activation of the MAPK pathway [22] and facilitated epithelial-mesenchymal transition (EMT) via an AKT pathway dependent on autophagy [23]. Using gene set enrichment analysis (GSEA) to examine transcriptional data from the TCGA BLCA cohort, we identified 14 significantly activated pathways in BCa samples, 130 upregulated pathways between RAB14-high and RAB14-low expression groups, and 11 overlapping pathways between the two comparisons (Fig. S1A). Notably, three sugar metabolism pathways were simultaneously activated in both the BLCA and RAB14-high groups (Fig. S1B–D). Previous RAB14 RNA-seq data identified 1049 dysregulated genes upon RAB14 knockdown in 5637 cells. Intersecting these genes with the core genes of the three identified sugar metabolism pathways yielded two candidates: PGM3 and GANAB (Fig. S1E). However, the role of GANAB in BCa has already been reported. The TIMER 2.0 database showed a significant correlation between PGM3 and RAB14 (Fig. S1F). Therefore, we speculated that PGM3 may be involved in the tumorigenesis and progression in BCa.

### PGM3 is highly expressed in BCa and correlates with poor prognosis

**Fig. 1:**
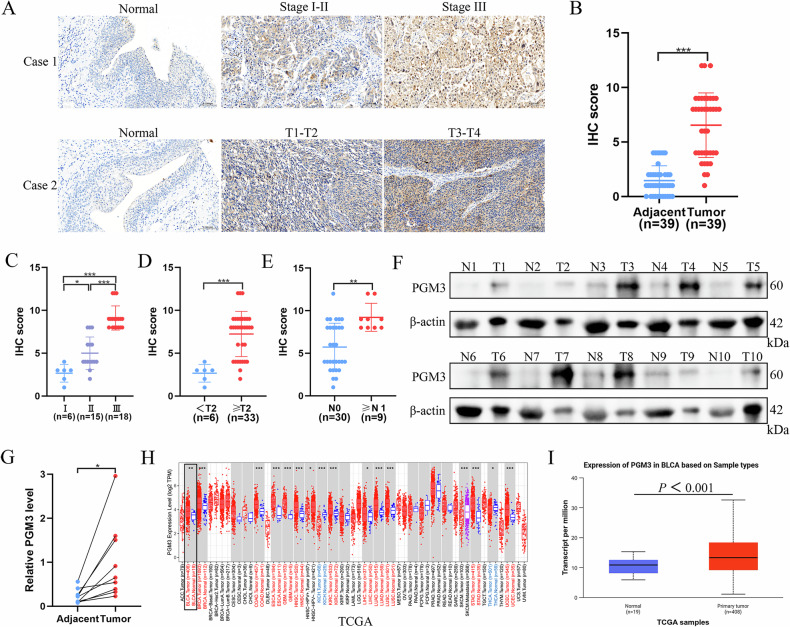
PGM3 is highly expressed in BCa and correlates with poor prognosis. The representative images of IHC staining for PGM3 between BCa tissues and their paired adjacent normal tissues (**A**) and IHC score based on IRS scoring system (*n* = 39) (**B**). The IHC score of PGM3 in different clinical stages (**C**), T stages (**D**), N stages (**E**) based on AJCC in BCa. The protein level of PGM3 by western blot analysis between BCa tissues and their paired adjacent normal tissues (*n* = 10) (**F**) and quantification analysis (**G**). **H** The differential expression of PGM3 between tumor and normal tissues in Pan-cancer from the TIMER database. **I** The expression of PGM3 between BCa and normal tissues from UALCAN based on the TCGA BLCA cohort. Data are presented as mean ± SD, the differences between groups were compared by Student’s *t*-test, (*) represents *P* < 0.05, (**) represents *P* < 0.01, (***) represents *P* < 0.001.

To elucidate the significance of PGM3 in the clinical context of BCa, we conducted IHC assessments of PGM3 levels in 39 matched sets comprising tumor tissues and their adjacent normal counterparts. The findings clearly demonstrated significantly higher PGM3 expression in tumor samples (Fig. 1A, B). Moreover, elevated PGM3 expression correlated positively with advanced clinical stage, T stage, and N stage (Fig. 1C–E). WB results corroborated IHC data, showing increased PGM3 protein levels in BCa tissues (Fig. 1F, G). Analysis of public databases further confirmed elevated PGM3 expression in 12 cancer types, including BLCA, based on TCGA datasets (Fig. 1H, I). Subsequent analyses utilizing data from TCGA, GEO, and the UROMOL cohorts revealed that higher expression of PGM3 was correlated with increased tumor stage, advanced pathological grade, and elevated T stage (Fig. S2A, B). Additionally, elevated PGM3 expression levels were predictive of poorer overall survival (OS) in the TCGA, GSE13507, and GSE31684 datasets, as well as decreased disease-specific survival (DSS) in the TCGA cohort (Fig. S2C). These collective findings strongly support a contributory role for PGM3 in promoting BCa initiation and malignant progression.

### PGM3 promotes BCa malignant progression in vitro and in vivo

**Fig. 2:**
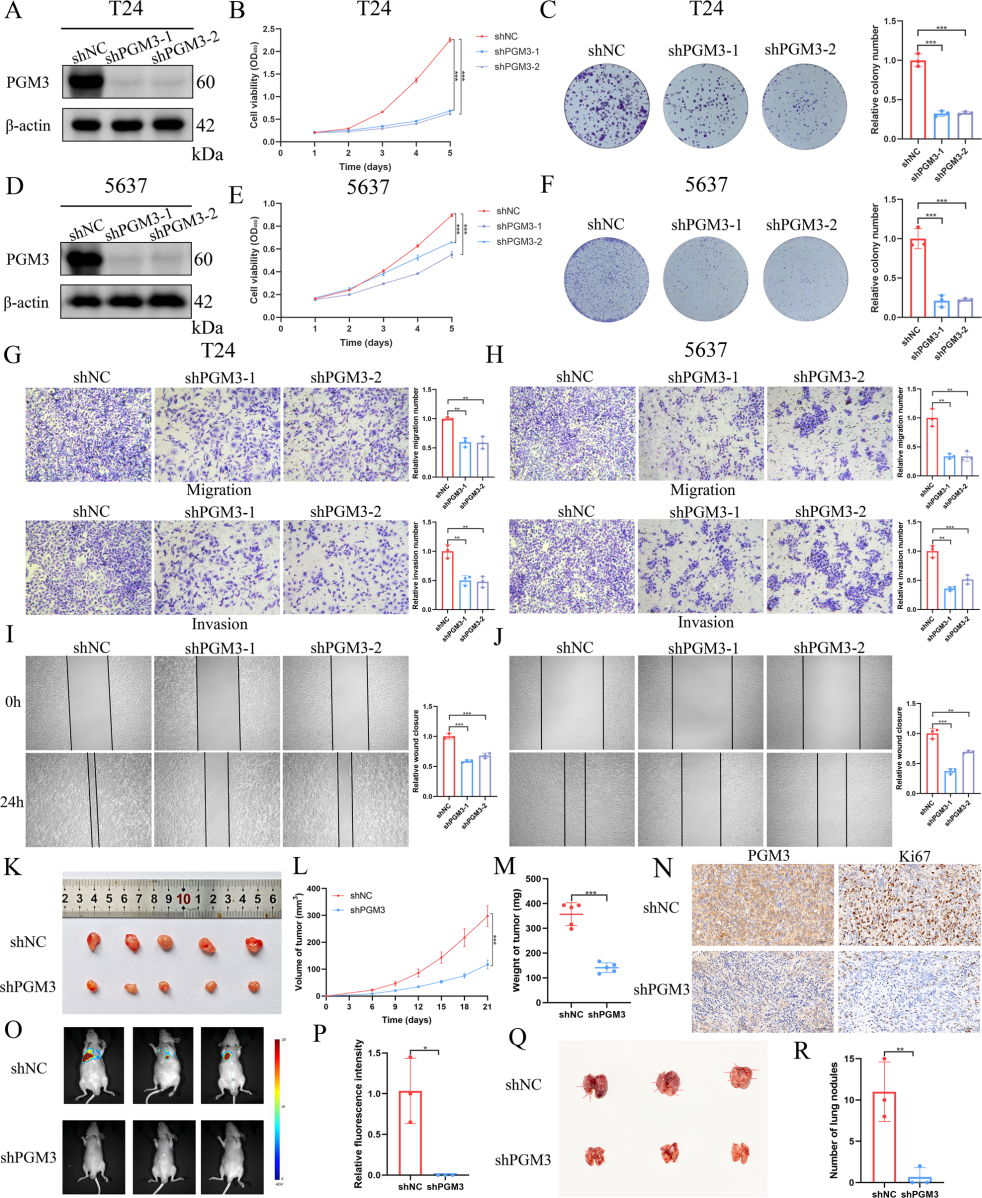
PGM3 promotes BCa malignant progression in vitro and in vivo. The knockdown efficiency validation by western blot in T24 (**A**) and 5637 cells (**D**) transfected with shPGM3-1 and shPGM3-2 lentiviruses. CCK-8 assays to determine the effect of PGM3 knockdown on cell proliferation in T24 (**B**) and 5637 cells (**E**). Colony formation assays to determine the effect of PGM3 knockdown on colony formation capability in T24 (**C**) and 5637 cells (**F**). Transwell assays to determine the influence of PGM3 knockdown on migration and invasion ability in T24 (**G**) and 5637 cells (**H**). Wound healing assays to determine the influence of PGM3 knockdown on migration ability in T24 (**I**) and 5637 cells (**J**). The comparisons of tumor volume (**K**), tumor growth rate (**L**) and tumor weight (**M**) in BALB/c nude mice injected with shNC and shPGM3 T24 cells. **N** Representatives images of IHC staining for PGM3 and Ki67 of tumor tissues in the xenograft model of nude mice. Fluorescence imaging (**O**), fluorescence intensity quantification (**P**), lung nodules graphs (**Q**) and lung nodules quantification (**R**) in lung metastasis model. Data are presented as mean ± SD, the differences between groups were compared by Student’s *t*-test, (*) represents *P* < 0.05, (**) represents *P* < 0.01, (***) represents *P* < 0.001.

To further define the biological roles of PGM3 in BCa, we designed two specific shRNAs targeting PGM3 (shPGM3-1 and shPGM3-2), and transduced them into T24 and 5637 cell lines. WB verified efficient reduction of PGM3 protein levels following shRNA treatment (Fig. 2A, D). Cellular proliferation, measured by CCK-8 assays, was significantly suppressed upon PGM3 knockdown (Fig. 2B, E). These results were further validated through colony formation assays (Fig. 2C, F). Moreover, wound healing and transwell assays uniformly showed that depletion of PGM3 notably reduced the migratory and invasive capabilities of T24 and 5637 cell lines (Fig. 2G–J). In vivo validation using nude mice xenograft models showed reduced tumor volumes, slower growth rates, and decreased tumor weights in mice injected with shPGM3 cells (Fig. 2K–M). IHC analysis revealed significantly reduced Ki67 expression following PGM3 depletion (Fig. 2N). The lung metastasis model indicated that the shPGM3 group had lower lung fluorescence intensity and fewer lung nodules than shNC group (Fig. 2O–R). These results indicate that PGM3 promotes BCa tumor growth and lung metastasis both in vitro and in vivo.

### PGM3 depletion inhibits BCa malignant progression by downregulating energy metabolism

**Fig. 3:**
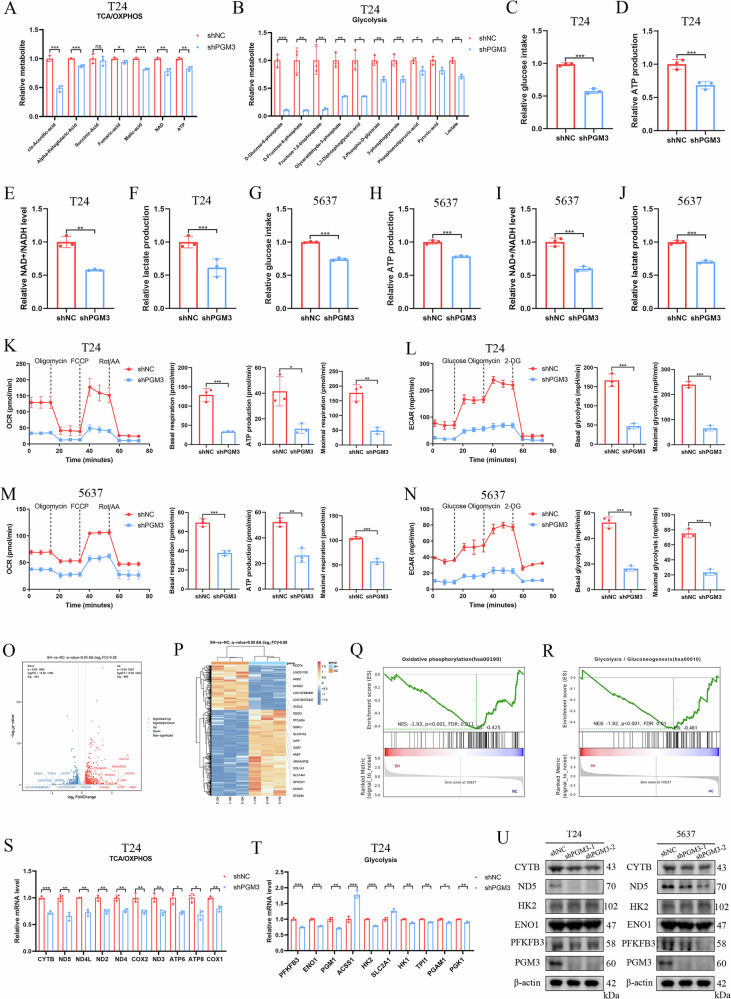
PGM3 depletion inhibits BCa malignant progression by downregulating energy metabolism. Metabolomic analysis to detect the metabolite change of TCA/OXPHOS (**A**) and glycolysis (**B**) pathways after PGM3 depletion compared with the shNC group in T24 cells. The assays of glucose uptake, ATP production, NAD^+^/NADH ratio, and lactate level by commercial kits in T24 (**C**–**F**) and 5637 cells (**G**–**J**). OCR and ECAR analysis to investigate the oxidative phosphorylation activity and glycolytic activity between the shPGM3 and shNC groups in T24 (**K**, **L**) and 5637 cells (**M**, **N**). Volcano map (**O**) and heatmap (**P**) of DEGs for RNA-seq after PGM3 knockdown in T24 cells. GSEA indicated that the OXPHOS pathway (**Q**) and the glycolysis/gluconeogenesis pathway (**R**) were significantly enriched after PGM3 depletion in T24 cells. Top ten genes of DEGs in the OXPHOS pathway (**S**) and glycolysis/gluconeogenesis pathway (**T**). **U** The protein level change by western blot of some key molecules in the OXPHOS pathway and glycolysis pathway in T24 and 5637 cells. Data are presented as mean ± SD, the differences between groups were compared by Student’s *t-*test, (*) represents *P* < 0.05, (**) represents *P* < 0.01, (***) represents *P* < 0.001.

Since PGM3 is a key enzyme in sugar metabolism pathways, we performed metabolite analyses after depleting PGM3 in T24 cells to investigate its regulatory mechanisms in BCa progression. PGM3 depletion significantly decreased metabolite levels in the TCA/OXPHOS pathway (Fig. 3A). Notably, PGM3 depletion also markedly reduced glycolytic metabolites (Fig. 3B). Additionally, glucose uptake, ATP production, NAD+/NADH ratios, and lactate production were significantly decreased in shPGM3-treated T24 and 5637 cells (Fig. 3C–J), further confirming that PGM3 regulates both OXPHOS and glycolysis. Consistent with metabolite analysis, OCR and ECAR assays revealed significantly reduced mitochondrial OXPHOS activity and glycolytic capacity in PGM3-depleted cells compared with controls (Fig. 3K–N). Collectively, these findings show that PGM3 depletion suppresses BCa progression by downregulating energy metabolism via inhibition of OXPHOS and glycolysis.

To further explore how PGM3 depletion regulates energy metabolism, we conducted RNA-seq in T24 cells with or without PGM3 knockdown. Compared to controls, 344 significantly downregulated and 660 significantly upregulated genes were identified in PGM3-depleted cells (Fig. 3O, P). GSEA indicated significant enrichment of OXPHOS and glycolysis/gluconeogenesis pathways (Fig. 3Q, R). The top 10 DEGs associated with OXPHOS and glycolysis pathways are shown in Fig. 3S, T. WB further verified significantly decreased protein expression of key OXPHOS enzymes (CYTB, ND5) and glycolytic enzyme (PFKFB3) after PGM3 knockdown (Fig. 3U). To confirm whether PGM3 promotes BCa progression via OXPHOS and glycolysis, we conducted functional experiments using specific pathway inhibitors: rotenone (OXPHOS inhibitor) and PFK-158 (PFKFB3 inhibitor). CCK-8 and colony formation assays showed that rotenone or PFK-158 treatment partially reversed the proliferative effects induced by PGM3 overexpression in T24 cells (Fig. S3A, B). Moreover, transwell and wound healing assays indicated that rotenone or PFK-158 treatment partially reduced the enhanced migratory and invasive abilities mediated by PGM3 overexpression (Fig. S3C, D). In xenograft experiments, administration of rotenone or PFK-158 significantly decreased tumor growth rates, tumor volumes, and tumor weights in mice injected with T24 cells overexpressing PGM3 (Fig. S3E. G). Collectively, these findings clearly demonstrate that suppression of PGM3 expression impedes BCa malignant progression by modulating energy metabolism pathways.

### PSMD11 interacts with and attenuates ubiquitination and degradation of PGM3

**Fig. 4:**
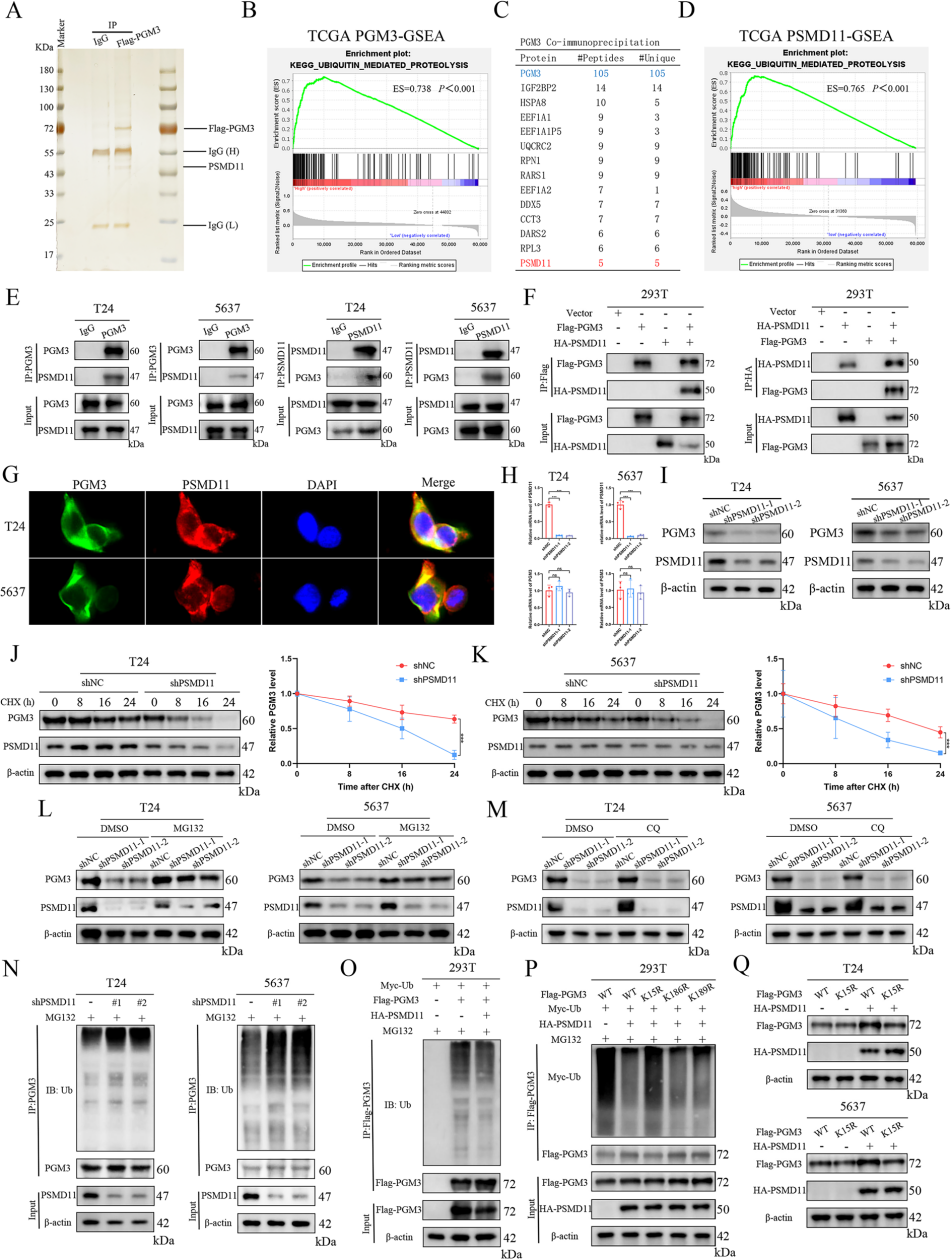
PSMD11 interacts with and attenuates ubiquitination and degradation of PGM3. **A** Silver staining of Co-IP samples for PGM3 in T24 cells. **B** GSEA of PGM3 based on TCGA BLCA cohort data. **C** The candidate proteins interacting with PGM3 identified by LC-MS analysis. **D** GSEA of PSMD11 based on TCGA BLCA cohort data. **E** Validation of interaction between endogenous PGM3 and PSMD11 by Co-IP in T24 and 5637 cells. **F** Validation of interaction between ectopically expressed Flag-PGM3 and HA-PSMD11 by Co-IP in 293 T cells. **G** Immunofluorescence analysis to determine the intracellular co-localization of PGM3 and PSMD11 in T24 and 5637 cells. RT-qPCR and western blot to determine the mRNA level (**H**) and protein level (**I**) change of PGM3 after PSMD11 knockdown in T24 and 5637 cells. Protein degradation assays by CHX (100 ug/ml) treatment for 0 h, 8 h, 16 h, and 24 h to explore the effect of PSMD11 depletion on the stability of PGM3 in T24 (**J**) and 5637 cells (**K**). Western blot analysis after proteasome inhibitor MG132 or lysosomal inhibitor CQ treatment for 8 h to confirm the influence of MG132 (**L**) and CQ (**M**) on PSMD11-mediated PGM3 regulatory effect in T24 and 5637 cells. **N** Endogenous ubiquitination level assays of PGM3 by Co-IP and WB after PSMD11 knockdown in T24 and 5637 cells. **O** Ubiquitination level assays of ectopically expressed PGM3 in 293 T cells co-transfected with HA-PSMD11 plasmid. **P** The effect of PSMD11 on the ubiquitination of PGM3 in 293 T cells transfected with WT PGM3, K15R, K186R, and K189R PGM3 mutant plasmids. **Q** Validation of the regulatory effect of PSMD11 on K15R PGM3 and WT PGM3 in T24 and 5637 cells.

Subsequently, to identify crucial modulators of PGM3 protein, we utilized immunoprecipitation (IP) followed by LC-MS in T24 cells (Fig. 4A). GSEA of PGM3-related expression signatures in the TCGA BLCA cohort indicated significant enrichment of the KEGG pathway linked to ubiquitin-mediated proteolysis within the group exhibiting high PGM3 expression (Fig. 4B). This suggested a potential regulatory mechanism involving the ubiquitin-proteasome pathway. Among the proteins identified as potential interactors of PGM3, the 26S proteasome regulatory non-ATPase subunit 11 (PSMD11), directly implicated in ubiquitin-proteasome system function, emerged as the most prominent interactor (Figs. 4C, S4A). Further GSEA in the TCGA BLCA cohort also confirmed heightened ubiquitin-mediated proteolysis signaling associated with elevated PSMD11 expression (Fig. 4D). Co-immunoprecipitation experiments validated the endogenous binding between PGM3 and PSMD11 proteins within T24 and 5637 cells (Fig. 4E). Additionally, exogenous Flag-PGM3 interacted with exogenous HA-PSMD11 in HEK293T cells (Fig. 4F). Immunofluorescence assays confirmed cytoplasmic co-localization of PGM3 and PSMD11 (Fig. 4G).

To determine the effect of PSMD11 on PGM3, WB and RT-qPCR analyses were performed. PSMD11 silencing significantly reduced PGM3 protein levels but not mRNA expression in T24 and 5637 cells (Fig. 4H, I). Conversely, PGM3 depletion did not alter PSMD11 protein levels (Fig. S4B), suggesting PSMD11 regulates PGM3 at the post-transcriptional level. Cycloheximide (CHX) chase assays demonstrated that PSMD11 deficiency accelerated PGM3 degradation, shortening its half-life (Fig. 4J, K). To clarify the degradation mechanism, shNC and shPSMD11 cells were treated with the proteasome inhibitor MG132 or lysosomal inhibitor chloroquine (CQ) for 8 hours. Immunoblotting indicated that MG132 treatment prevented PGM3 degradation induced by PSMD11 knockdown, while CQ had no effect (Fig. 4L, M). Thus, PSMD11 depletion promotes PGM3 degradation via the ubiquitin-proteasome pathway. Furthermore, endogenous and exogenous ubiquitination assays demonstrated that PSMD11 knockdown significantly increased PGM3 ubiquitination, whereas PSMD11 overexpression reduced it (Fig. 4N, O). Collectively, PSMD11 stabilizes PGM3 by attenuating proteasomal degradation via reduced ubiquitination.

To identify the specific polyubiquitin chain type regulated by PSMD11, we expressed wild-type Myc-tagged ubiquitin (Myc-Ub WT) or single-lysine ubiquitin mutants in HEK293T cells. PSMD11 specifically inhibited K48-linked ubiquitination of PGM3 (Fig. S4C). Consistent findings were observed when lysine residues were mutated to arginine (K → R); PSMD11 increased ubiquitination only with the K48R mutant compared to WT ubiquitin (Fig. S4D). LC-MS/MS identified lysine residues on PGM3 potentially involved in ubiquitination (Table S4). Mutational analysis (K15R, K186R, K189R) indicated that PSMD11 specifically decreased ubiquitination at the K15 residue of PGM3 in 293T cells (Fig. 4P). This was validated by the fact that PSMD11 could only stabilize WT PGM3 rather than K15R PGM3 in T24 and 5637 cells (Fig. 4Q). Thus, PSMD11 inhibits K48-linked ubiquitination at K15, stabilizing PGM3.

**Fig. 5:**
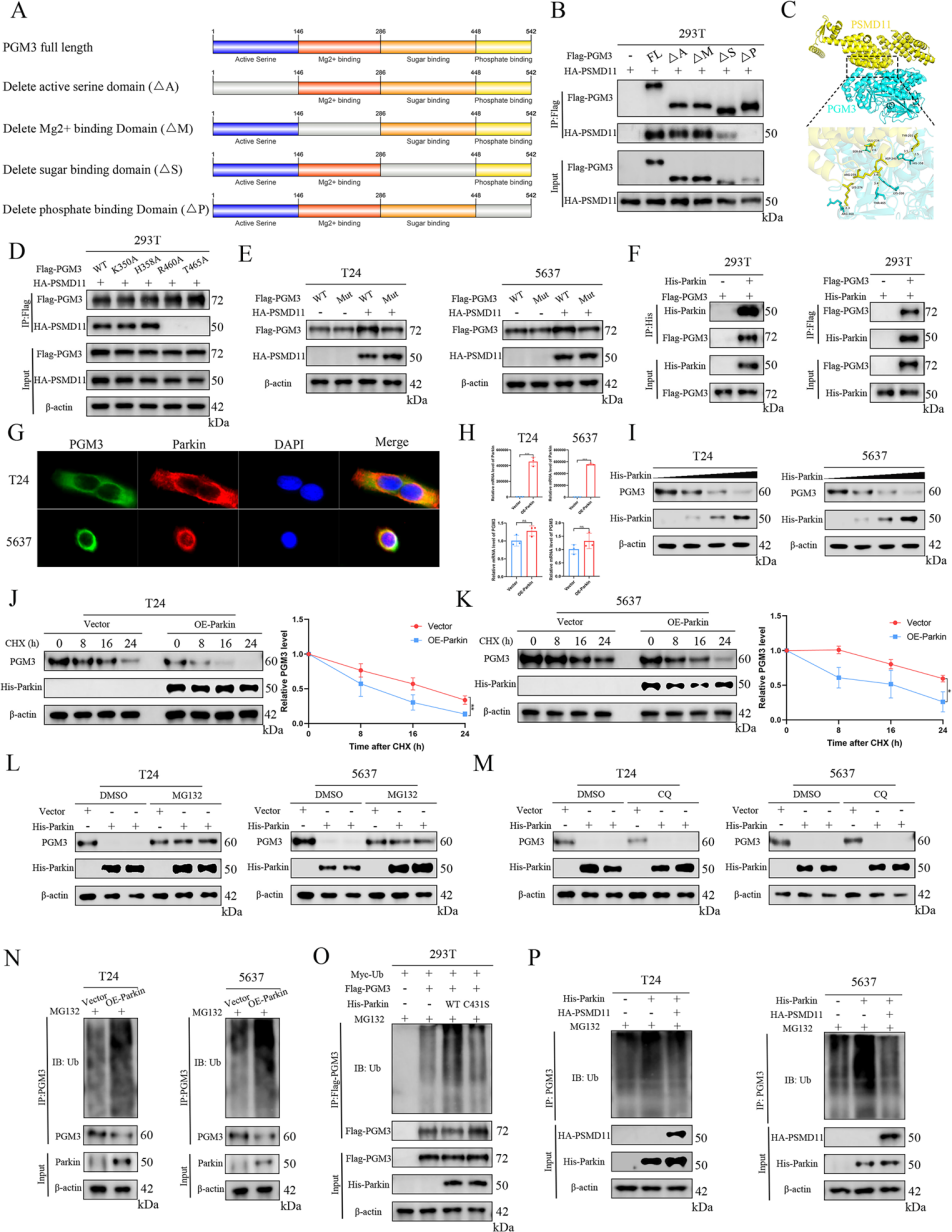
PSMD11 reduces PGM3 ubiquitination by antagonizing Parkin-mediated ubiquitination. **A** The structure schematic diagram of full-length PGM3 and PGM3 domain-deletion mutants. **B** Co-IP and WB performed the interaction analysis between PSMD11 and full-length and domain-deletion mutant PGM3 in 293 T cells. **C** The molecular docking model between PSMD11 and PGM3. **D** Co-IP and WB performed the interaction analysis between PSMD11 and WT PGM3 and site-mutant PGM3, including K350A, H358A, R460A, and T465A in 293 T cells. **E** Validation of the regulatory effect of PSMD11 on R460A & T465A PGM3 and WT PGM3 in T24 and 5637 cells. **F** Validation of interaction between ectopically expressed Flag-PGM3 and His-Parkin by Co-IP and WB in 293 T cells. **G** Immunofluorescence analysis to determine the intracellular co-localization of PGM3 and Parkin in T24 and 5637 cells. RT-qPCR and western blot to determine the mRNA level (**H**) and protein level (**I**) change of PGM3 after Parkin overexpression in T24 and 5637 cells. Protein degradation assays by CHX (100 ug/ml) treatment for 0 h, 8 h, 16 h and 24 h to explore the effect of Parkin overexpression on the stability of PGM3 in T24 (**J**) and 5637 cells (**K**). Western blot analysis after proteasome inhibitor MG132 or lysosomal inhibitor CQ treatment for 8 h to confirm the influence of MG132 (**L**) and CQ (**M**) on Parkin-mediated PGM3 regulatory effect in T24 and 5637 cells. **N** Endogenous ubiquitination level assays of PGM3 by Co-IP and WB after Parkin overexpression in T24 and 5637 cells. **O** Ubiquitination level assays of ectopically expressed PGM3 in 293 T cells co-transfected with WT His-Parkin or C431S His-Parkin plasmids. **P** The effect of PSMD11 overexpression on Parkin-mediated ubiquitination of PGM3 in T24 and 5637 cells.

To define the PGM3 domain interacting with PSMD11, domain-deletion mutants of PGM3 were generated and transfected into HEK293T cells (Fig. 5A). Co-IP assays showed that PSMD11 interacted with all domain mutants except the phosphate-binding domain deletion (residues 448–542), indicating that this region is necessary for PSMD11-PGM3 interaction (Fig. 5B). Molecular docking identified potential interaction residues (K350, H358, R460, T465) between PGM3 and PSMD11, which formed good hydrogen bonding sites with PSMD11 (Fig. 5C). Alanine substitutions (K350A, H358A, R460A, T465A) demonstrated that mutations at R460 and T465 abolished binding with PSMD11 (Fig. 5D), which was consistent with the finding that PSMD11 interacts with PGM3 at amino acid residues 448-542. Further, combined mutation (R460&T465) prevented PSMD11-mediated stabilization of PGM3 in T24 and 5637 cells (Fig. 5E), confirming these residues as critical for interaction.

Together, these data indicate that PSMD11 binds the phosphate-binding domain of PGM3 and stabilizes it by reducing K48-linked polyubiquitination at K15.

### PSMD11 reduces PGM3 ubiquitination by antagonizing Parkin-mediated ubiquitination

Interestingly, PSMD11 lacks known E3 ubiquitin ligase or deubiquitinating enzyme (DUB) activity. Thus, we speculated that PSMD11 indirectly modulates PGM3 ubiquitination by affecting other ubiquitin ligases or DUBs targeting PGM3. No known ubiquitin ligases or DUBs for PGM3 were reported. However, analysis of the BioGRID and HitPredict databases revealed that the top-ranked interacting protein of PGM3, Parkin, is an E3 ubiquitin ligase. Co-IP confirmed exogenous interactions between PGM3 and Parkin in 293 T cells and endogenous interactions in T24 and 5637 cells (Figs. 5F, S4E). IF assays confirmed cytoplasmic co-localization of PGM3 and Parkin (Fig. 5G).

Next, we determined whether Parkin is the E3 ubiquitin ligase regulating PGM3 expression. Parkin overexpression decreased PGM3 protein levels dose-dependently without altering PGM3 mRNA in T24 and 5637 cells (Fig. 5H, I). Parkin also accelerated PGM3 protein degradation in T24 and 5637 cells (Fig. 5J, K). Proteasome inhibition (MG132) reversed Parkin-mediated PGM3 degradation, while lysosomal inhibition (CQ) had no effect (Fig. 5L, M), confirming proteasome-dependent degradation. Both endogenous and exogenous ubiquitination assays indicated that Parkin significantly enhanced PGM3 ubiquitination, dependent on its catalytic activity (C431) (Fig. 5N, O). Additionally, ubiquitin mutant assays confirmed Parkin specifically catalyzed K48-linked ubiquitination of PGM3 (Fig. S4G, H).

Further, we evaluated whether PSMD11 antagonizes Parkin-mediated ubiquitination. The results revealed that PSMD11 overexpression significantly reversed Parkin-induced PGM3 ubiquitination in endogenous and exogenous validation (Figs. 5P, S4F). Mechanistically, PSMD11 reduced Parkin binding to PGM3 without affecting Parkin expression levels in 293 T, T24, and 5637 cells (Fig. S4I, J), indicating competitive binding between PSMD11 and Parkin. Molecular docking showed both proteins interact with PGM3 at residues R460 and T465 (Figs. 5C, S4K). Domain-mapping assays confirmed Parkin binds to the same 448-542 region of PGM3 as PSMD11 (Fig. S4L). Therefore, PSMD11 competes with Parkin for binding to PGM3, attenuating Parkin-mediated ubiquitination.

### PSMD11 protein is upregulated in BCa tumors and positively correlated with PGM3 expression

**Fig. 6:**
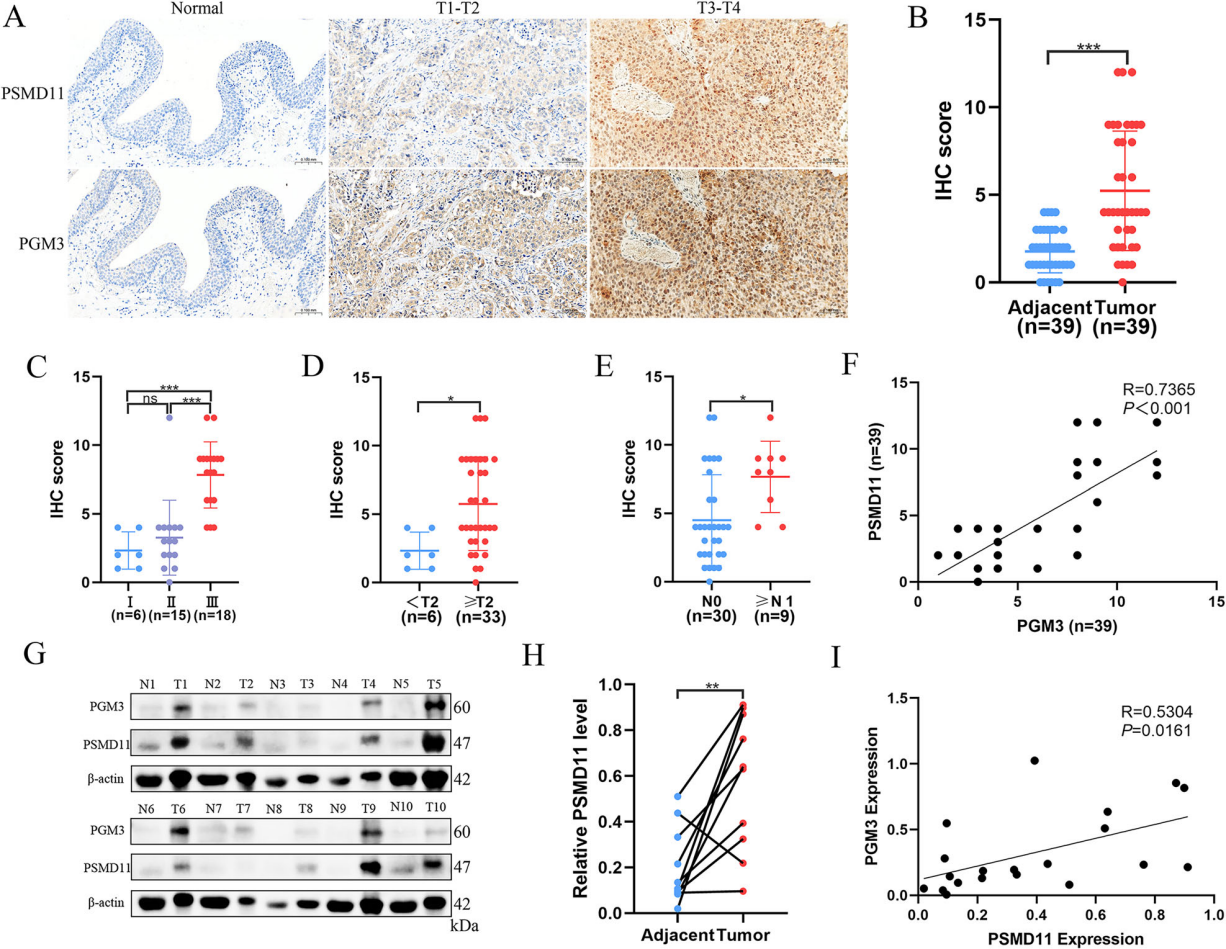
PSMD11 protein is upregulated in BCa tumors and positively correlated with PGM3 expression. The representative images of IHC staining for PSMD11 between BCa tissues and their paired adjacent normal tissues (**A**) and IHC score based on IRS scoring system (*n* = 39) (**B**). The IHC score of PSMD11 in different clinical stages (**C**), T stages (**D**), N stages (**E**) based on AJCC in BCa. **F** Correlation analysis between PSMD11 and PGM3 based on IHC score. The protein level of PSMD11 by western blot analysis between BCa tissues and their paired adjacent normal tissues (*n* = 10) (**G**) and quantification analysis (**H**), and the correlation analysis of PSMD11 and PGM3 based on protein level (**I**). Data are presented as mean ± SD, the differences between groups were compared by student’s *t* test, (*) represents *P* < 0.05, (**) represents *P* < 0.01, (***) represents *P* < 0.001.

The clinical implications of PSMD11 expression in BCa were also examined. Immunohistochemistry results revealed substantially higher PSMD11 protein expression in tumor specimens (Fig. 6A, B). Additionally, increased PSMD11 expression correlated positively with higher clinical stage, advanced T stage, and lymph node involvement (Fig. 6C–E). Patients with high PSMD11 expression also exhibited elevated PGM3 expression, indicating a positive correlation (Fig. 6A, F). WB results supported IHC findings, demonstrating PSMD11 overexpression and its positive correlation with PGM3 levels in BCa (Fig. 6G–I). Additionally, analyses of public databases confirmed that PSMD11 was upregulated in 12 cancers, including BLCA in the TCGA dataset (Fig. S5A, B), a finding further validated in the GSE13507 cohort (Fig. S5B). Analysis of TCGA, GEO databases, and the UROMOL cohort indicated that high PSMD11 expression correlated significantly with pathological grade and T stage (Fig. S5C, D). Furthermore, elevated PSMD11 expression predicted poor OS (TCGA, GSE13507, GSE48075) and DSS (TCGA) in BCa patients (Fig. S5E). Collectively, these findings indicate that PSMD11 is upregulated in BCa and positively associated with PGM3 expression.

### PSMD11 enhances energy metabolism to promote BCa malignant progression via PGM3

**Fig. 7:**
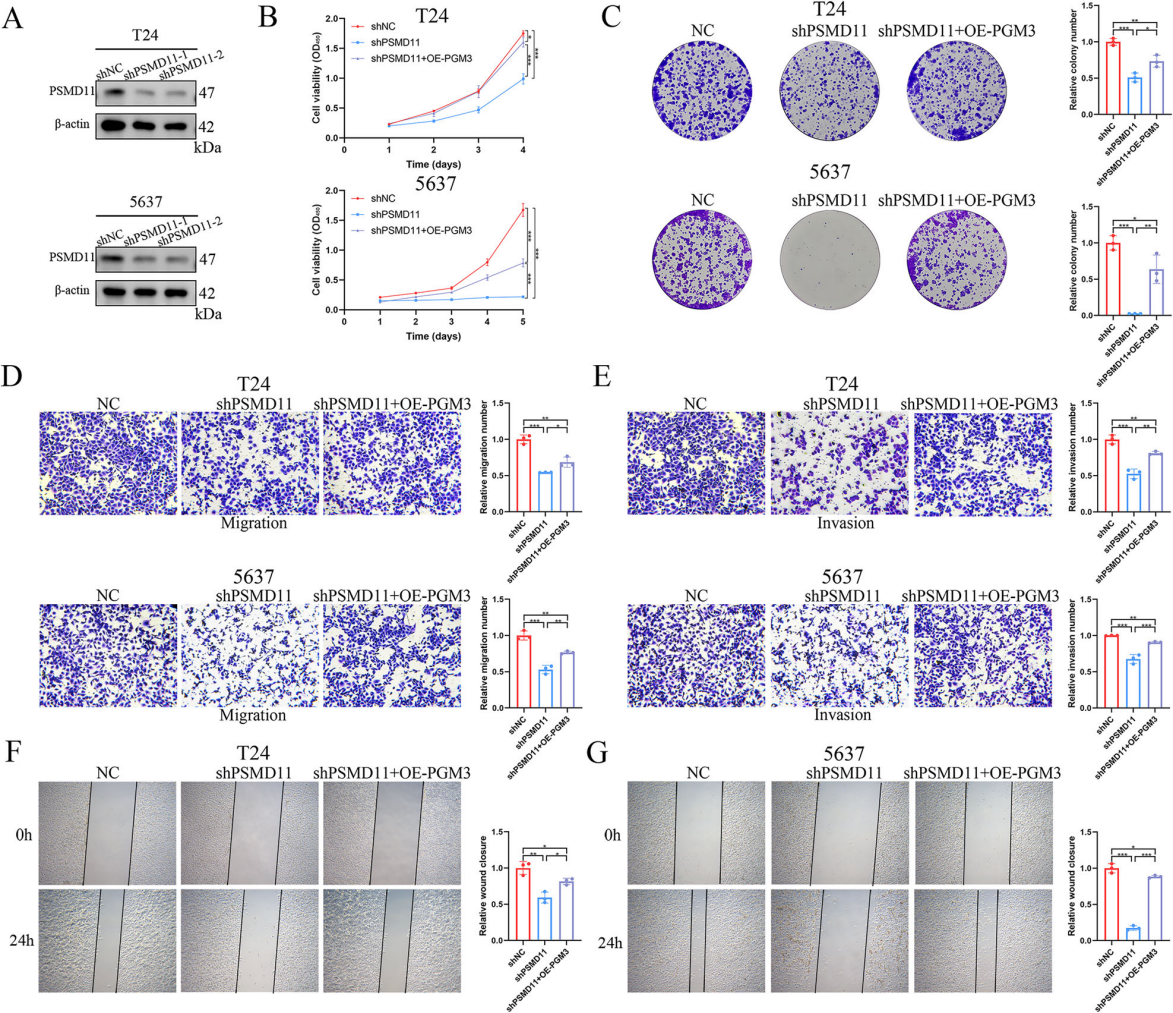
PSMD11 promotes BCa malignant progression in vitro via regulating PGM3. **A** The knockdown efficiency validation by western blot in T24 and 5637 cells transfected with shPSMD11-1 and shPSMD11-2 lentiviruses. CCK-8 (**B**) and colony formation (**C**) assays to determine the effect of PSMD11 knockdown and PSMD11 knockdown combined with PGM3 overexpression on cell proliferation in T24 and 5637 cells. Transwell (**D**, **E**) and wound healing (**F**, **G**) assays to determine the influence of PSMD11 knockdown and PSMD11 knockdown combined with PGM3 overexpression on migration and invasion ability in T24 and 5637 cells. Data are presented as mean ± SD, the differences between groups were compared by Student’s *t*-test, (*) represents *P* < 0.05, (**) represents *P* < 0.01, (***) represents *P* < 0.001.

**Fig. 8:**
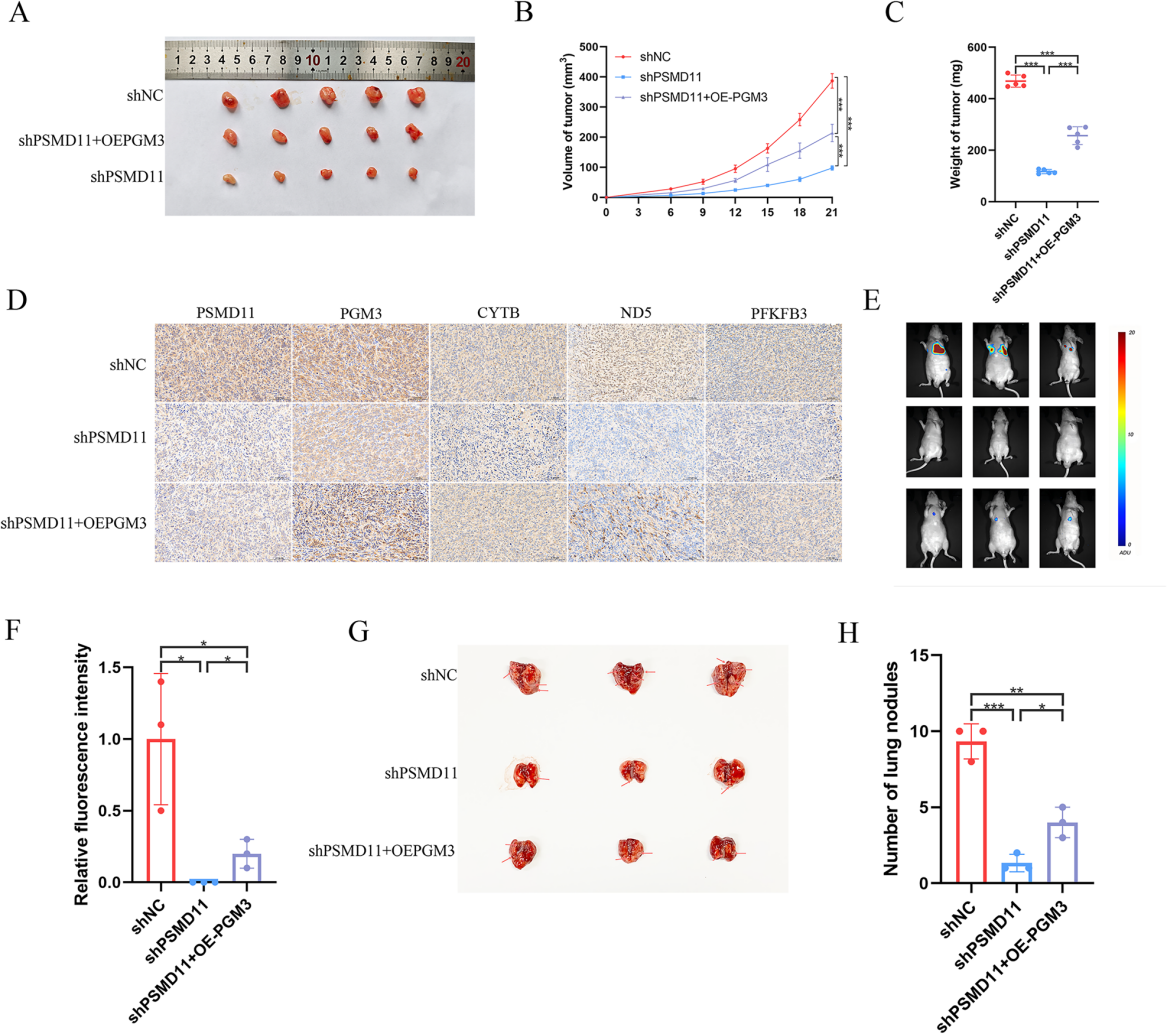
PSMD11 promotes BCa malignant progression in vivo via regulating PGM3. The comparisons of tumor volume (**A**), tumor proliferation rate (**B**), and tumor weight (**C**) in BALB/c nude mice injected with shNC, shPSMD11, and shPSMD11 combined with PGM3 overexpression T24 cells. **D** Representatives images of IHC staining for PSMD11, PGM3, CYTB, ND5, and PFKFB3 of tumor tissues in the xenograft model of nude mice. Fluorescence imaging (**E**), fluorescence intensity quantification (**F**), lung nodules graphs (**G**) and lung nodules quantification (**H**) in lung metastasis model. Data are presented as mean ± SD, the differences between groups were compared by Student’s *t*-test, (*) represents *P* < 0.05, (**) represents *P* < 0.01, (***) represents *P* < 0.001.

To elucidate the biological functions of PSMD11 in BCa, two independent PSMD11-shRNAs (shPSMD11-1 and shPSMD11-2) were designed and introduced into T24 and 5637 cells. WB confirmed successful knockdown of PSMD11 protein expression (Fig. 7A). We selected shPSMD11-1 to perform the subsequent experiment. Cellular proliferation assays, including CCK-8 and colony formation, indicated significant reductions in cell growth following PSMD11 knockdown, effects that were rescued upon overexpression of PGM3 (Fig. 7B, C). Additionally, transwell migration/invasion and wound-healing assays further demonstrated that depletion of PSMD11 impaired the invasive and migratory capacities of BCa cells, with these inhibitory effects reversed by PGM3 overexpression (Fig. 7D–G). In vivo experiments involved establishing a xenograft mouse model through subcutaneous injection of T24 cells transfected with shNC, shPSMD11, or shPSMD11 combined with PGM3 overexpression. Compared with control mice, tumor volume, proliferation rate, and tumor weight were substantially reduced following PSMD11 depletion, effects also reversed by elevated expression of PGM3 (Fig. 8A–C). IHC analysis confirmed significant reductions in the expression of PGM3, CYTB, ND5, and PFKFB3 proteins upon PSMD11 knockdown, reversed by PGM3 overexpression (Fig. 8D). Lung metastasis model indicated that the shPSMD11 group had lower lung fluorescence intensity and fewer lung nodules than the shNC group, this effect could be partly reversed by PGM3 overexpression (Fig. 8E–H). These findings indicate that PSMD11 promotes BCa progression by modulating PGM3 in vitro and in vivo.

**Fig. 9:**
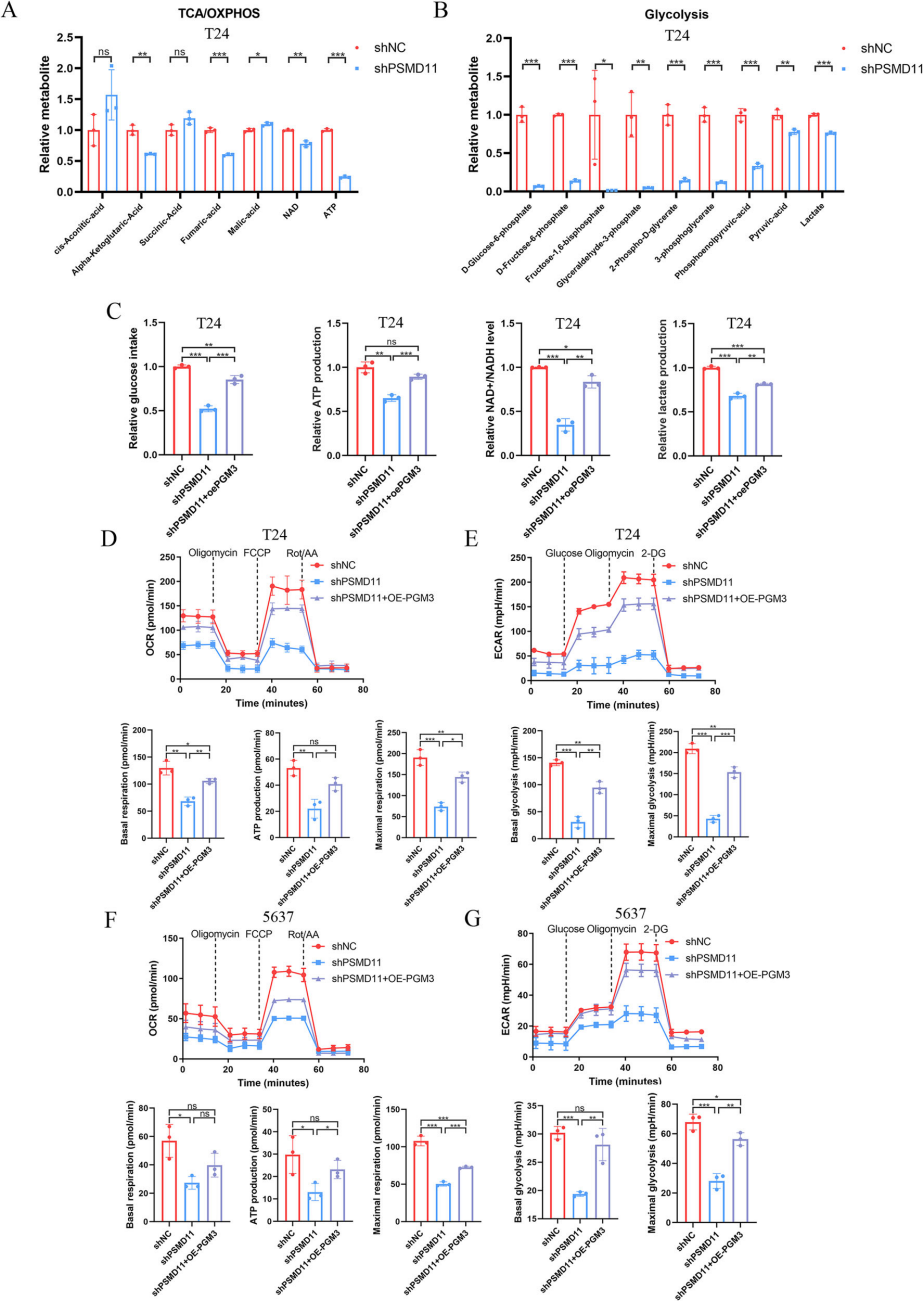
PSMD11 enhances energy metabolism to promote BCa malignant progression via PGM3. Metabolomic analysis to detect the metabolite change of TCA/OXPHOS (**A**) and glycolysis (**B**) pathways after PSMD11 depletion compared with the shNC group in T24 cells. **C** The assays of glucose uptake, ATP production, NAD^+^/NADH ratio, and lactate level by commercial kits in control, PSMD11 knockdown, and PSMD11 knockdown combined with PGM3 overexpression groups in T24 cells. OCR and ECAR analysis to investigate the oxidative phosphorylation activity and glycolytic activity between shNC, shPSMD11, and shPSMD11 combined with PGM3 overexpression groups in T24 (**D**, **E**) and 5637 cells (**F**, **G**). Data are presented as mean ± SD, the differences between groups were compared by Student’s *t*-test, (*) represents *P* < 0.05, (**) represents *P* < 0.01, (***) represents *P* < 0.001.

To further investigate whether PSMD11 affects energy metabolism via PGM3 in BCa, metabolite analyses were performed in PSMD11-depleted T24 cells. PSMD11 knockdown significantly reduced metabolite levels in both the TCA/OXPHOS and glycolytic pathways (Fig. 9A, B). Additionally, glucose uptake, ATP production, NAD⁺/NADH ratio, and lactate production significantly decreased upon PSMD11 depletion, and these reductions were rescued by PGM3 overexpression (Fig. 9C). Consistent with metabolite changes, OCR and ECAR assays showed that PSMD11 depletion significantly reduced mitochondrial OXPHOS and glycolytic activities, effects reversed by PGM3 overexpression (Fig. 9D–G). Altogether, these results demonstrate that PSMD11 enhances energy metabolism through PGM3, promoting malignant progression of BCa.

### PGM3 inhibitor suppresses BCa malignant progression

FR054, a selective inhibitor targeting the hexosamine biosynthesis pathway enzyme PGM3, has previously demonstrated potent antitumor effects against breast cancer [21]. To explore the therapeutic promise of pharmacologically targeting PGM3 in BCa, T24, and 5637 cells underwent treatment with FR054 (0.5 mM) for 24 hours. CCK-8 proliferation and colony-forming assays indicated that FR054 inhibited cell growth (Fig. S6A–D). Additionally, both wound healing and transwell assays demonstrated substantial impairment in cellular migration and invasion capabilities following FR054 administration (Fig. S6E, F). To examine FR054 efficacy in vivo, nude mice bearing subcutaneous T24 xenografts were administered FR054 (500 mg/kg/day, intraperitoneal injection) beginning six days post-inoculation. Tumors harvested from mice in the FR054 treatment group were significantly reduced in size compared to tumors from vehicle-treated control mice (Fig. S6G). Furthermore, FR054 significantly reduced tumor weight (Fig. S6H) and growth rates (Fig. S6I). These results collectively confirm that FR054 effectively inhibits malignant BCa progression in vitro and in vivo.

## Discussion

Despite accumulating evidence implicating PGM3 as a critical regulator in HBP and related carbohydrate metabolism pathways, current research into the functions of PGM3 remains insufficient. Previous studies have linked mutations in PGM3 with congenital disorders of glycosylation (CDG), resulting in severe immune deficiency, skeletal abnormalities, autoimmune disorders, and impaired cognitive development [25–27]. Collectively, these observations emphasize the indispensable role of PGM3 in regulating normal physiological processes, with emerging evidence reinforcing its pivotal contribution to cancer development and progression. The selective PGM3 inhibitor FR054 demonstrated substantial anti-tumor activity in breast cancer via inhibition of both N- and O-glycosylation [18]. Previous studies have associated heightened PGM3 expression with resistance to gemcitabine in pancreatic tumors; notably, administration of FR054 compromised protein glycosylation and elicited a persistent unfolded protein response (UPR), culminating in cancer cell apoptosis [19]. Analogously, KRAS/LKB1 double-mutant lung cancer cells demonstrated heightened reliance on PGM3 activity, and inhibition of this enzyme substantially reduced tumor proliferation both in vitro and within animal model systems [20]. Additionally, blocking PGM3 in glioblastoma reduced hexosamine pathway flux, suppressed expression of HBP enzymes, and markedly inhibited tumor proliferation [21]. Nevertheless, the exact contribution of PGM3 to BCa has remained unclear. Our present study reveals elevated PGM3 expression in BCa tissues, its association with unfavorable patient prognosis, and the ability of PGM3 inhibition to effectively restrain tumor progression. Moreover, the pharmacological agent FR054 exhibited robust efficacy in reducing BCa progression.

PGM3 functions as a pivotal enzyme in the HBP; thus, prior investigations predominantly focused on elucidating its role in tumor development via modulation of glycosylation and O-GlcNAcylation processes [18–21, 28]. Nevertheless, considering the inherent heterogeneity of tumors, PGM3 may influence cancer progression through additional, unexplored metabolic routes. Notably, PGM3 belongs to the phosphoglucomutase enzyme family, which mediates crucial steps in glucose metabolism, specifically facilitating the reversible conversion between G-1-P and G-6-P. Previous studies reported that PGM1 orchestrated metabolic reprogramming via glycolysis and OXPHOS, exerting pro- or anti-tumorigenic effects across various cancers [29–34]. Our metabolomics data demonstrated that PGM3 depletion significantly reduced metabolites associated with glycolysis and OXPHOS, confirmed by OCR and ECAR assays. RNA sequencing indicated decreased expression of key OXPHOS and glycolysis-related enzymes. Protein levels of CYTB, ND5, and PFKFB3 decreased upon PGM3 inhibition. Importantly, the OXPHOS inhibitor rotenone and glycolysis inhibitor PFK-158 reversed the tumor-promoting effects of PGM3 overexpression in BCa cells. Alternatively, emerging studies suggest that O-GlcNAcylation by OGT in HBP may influence tumor progression through glycolysis and OXPHOS [35–38]. Nevertheless, whether PGM3 directly regulates these metabolic pathways or acts indirectly through O-GlcNAcylation remains unclear.

Considering the pivotal role of energy metabolism reprogramming in carcinogenesis, current pharmacological strategies focus on targeting key glycolytic enzymes and kinases. These approaches disrupt enhanced glycolytic activity, initially restore mitochondrial respiration, and ultimately induce apoptosis [9–11]. However, therapeutic efficacy is limited by metabolic plasticity, as tumor cells maintain functional mitochondria. This adaptation allows malignant cells to switch from glycolysis to OXPHOS-derived ATP production, overcoming metabolic stress and supporting survival [12–15]. Recent evidence suggested that combined targeting of aerobic glycolysis and OXPHOS represents a more effective cancer therapy [39–41]. Our study provides a novel therapeutic strategy that genetically inhibits both glycolytic and OXPHOS pathways concurrently, thereby suppressing tumor progression.

Despite its significant regulatory role in glucose metabolism, the exact molecular pathways controlling PGM3 expression and catalytic activity remain poorly defined. GSEA indicated a potential link between PGM3 expression and activation of the ubiquitin-proteasome system (Fig. 4B). Using LC-MS, we identified PSMD11 as a potential regulator and confirmed that PSMD11 modulated PGM3 at the post-translational level, affecting protein abundance without altering mRNA expression. Additional experiments revealed that PSMD11 stabilized PGM3 by reducing K48-linked polyubiquitination at the K15 residue, functioning similarly to DUB. Previous studies indicated that PSMD14, another PSMD family member, possesses deubiquitinase activity and exerts pro-tumor effects [42–45]. However, other PSMD members, including PSMD11, lack intrinsic DUB activity [46]. Notably, post-translational modifications of PGM3 may influence its catalytic activity. Therefore, the regulatory effects of PSMD11 and Parkin might not be limited to protein stability (non-classical pathway) but may also involve modulation of enzymatic activity (classical pathway), ultimately influencing glycolysis, OXPHOS, and BCa progression.

Since PSMD11 is neither an E3 ligase nor a DUB, we speculated that an intermediate E3 ligase or DUB may exist between PSMD11 and PGM3. We searched the BioGRID and HitPredict databases, identifying Parkin (an E3 ubiquitin-protein ligase) as the top interacting partner of PGM3. Further experiments confirmed that Parkin promoted ubiquitination and proteasomal degradation of PGM3 in an enzyme activity-dependent manner, indicating Parkin is a specific E3 ligase for PGM3. Domain-deletion mutant analyses revealed that PSMD11 and Parkin competitively bind to the same region of PGM3, which molecular docking further supported. Parkin is a key regulator of mitochondrial quality control [47]; its role in Parkinson’s disease is established, but its involvement in cancer has recently emerged [48]. Parkin influences tumorigenesis and cancer progression via mitophagy in various cancers, including BCa [49–52]. However, Parkin substrates involved in mitophagy in BCa remain unidentified. Our results indicate that PGM3 is a novel substrate of Parkin, and that PGM3 regulates the expression of mitochondrial-encoded proteins (CYTB, ND5). Consequently, this interaction could lead to mitochondrial dysfunction and mitophagy, although these hypotheses require further experimental validation.

## Conclusions

**Fig. 10:**
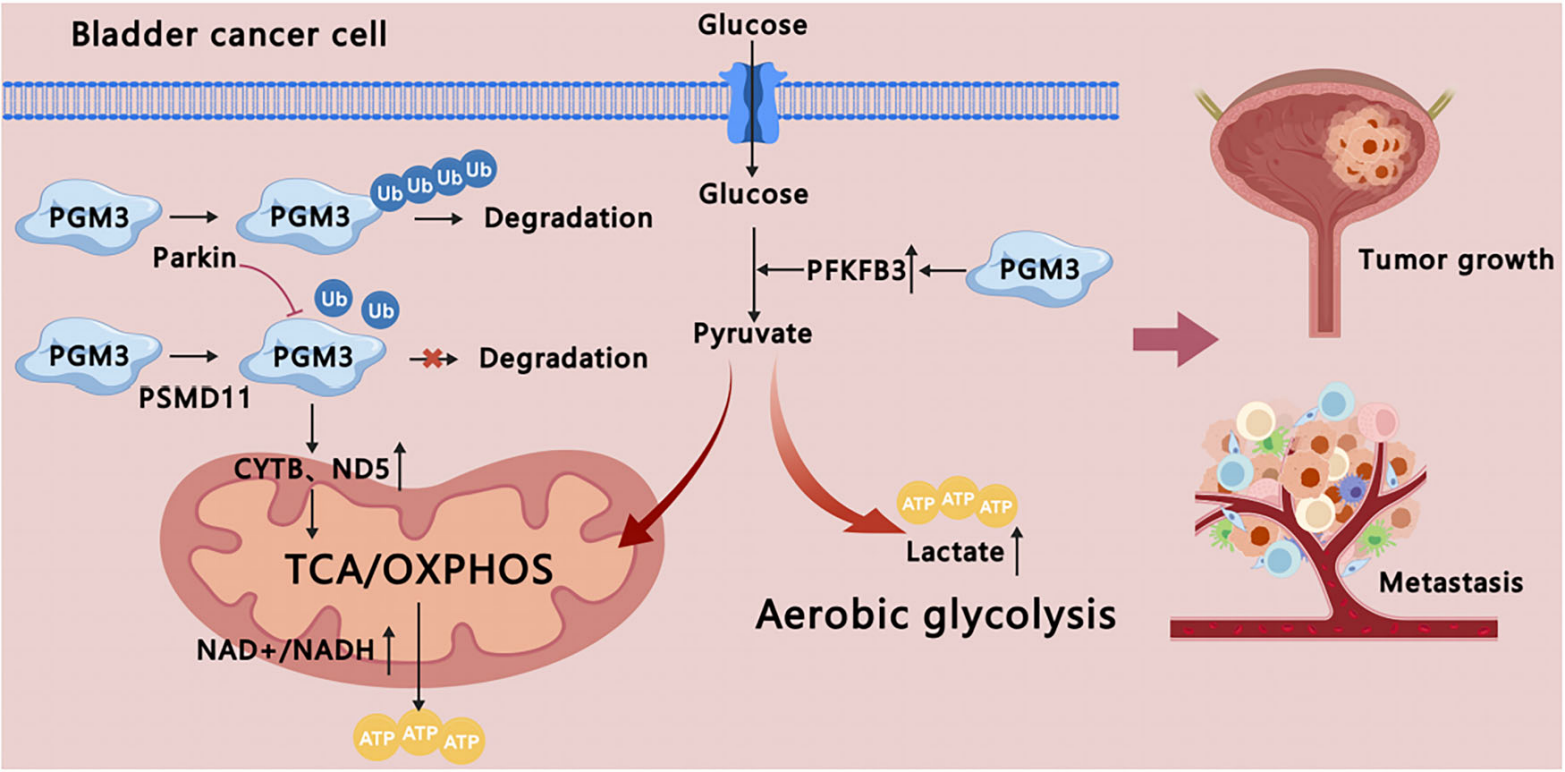
The schematic diagram of the mechanism proposed in this study.

In this study, we found that PGM3 was markedly overexpressed and associated with unfavorable prognosis in BCa. PGM3 promoted BCa malignant progression through glycolysis and OXPHOS. Mechanistically, PSMD11 competed with Parkin for PGM3 binding, thereby attenuating Parkin-mediated ubiquitination and stabilizing PGM3 (Fig. 10). These findings suggest that targeting the PSMD11/PGM3 axis could provide a promising therapeutic strategy for BCa.

## Supplementary information

Supplementary figures

Supplementary tables

uncropped gels and blots images

## Data Availability

The transcription profiles of BCa patients were retrieved from the TCGA database. BLCA gene microarray expression profiles (GSE13507, GSE31684, GSE32548, GSE32894, and GSE48075) were obtained from the GEO database. The publicly accessible UROMOL cohort data were downloaded from ArrayExpress. Pan-cancer analyses of PGM3 and PSMD11 were performed using the TIMER database. Prognostic information for PGM3 and PSMD11 was acquired from PanCanSurvPlot. The datasets used and/or analyzed during the current study are available from the corresponding author on reasonable request.
