# Supplementary material for: PSMD11 stabilizes PGM3 by antagonizing Parkin to promote bladder cancer progression through energy metabolism reprogramming
Source: Cell Death Dis. 2026 Apr 6;17(1):457. doi: 10.1038/s41419-026-08691-4 (PMC13184242; doi:10.1038/s41419-026-08691-4)
Supplement: Supplementary file 1 — Supplementary figures [file 41419_2026_8691_MOESM1_ESM.pdf]

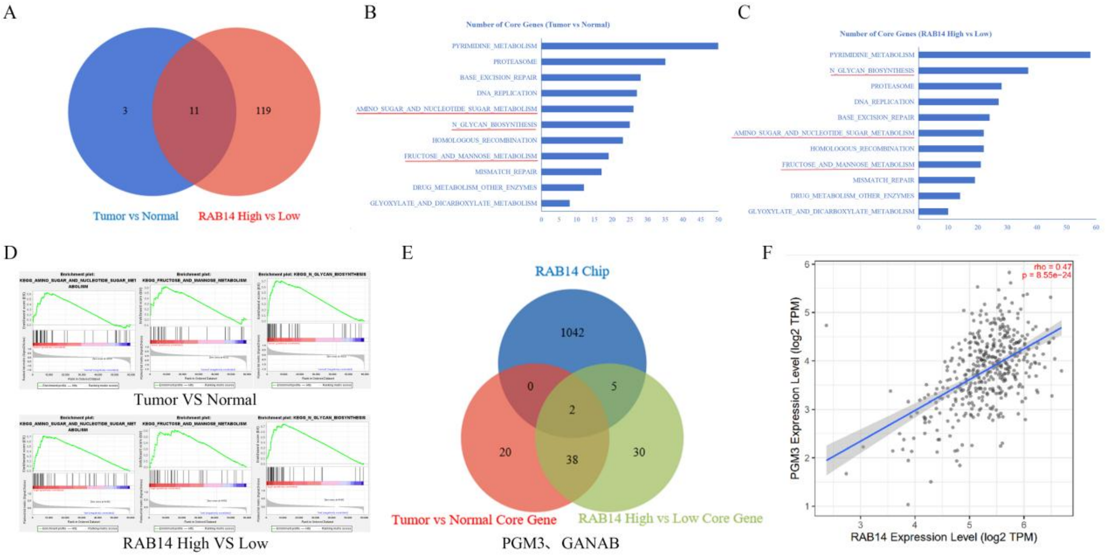

Figure S1. A GSEA analysis between BCa group and normal group (blue) and between RAB14 high expression group and low expression group (red) based on TCGA BLCA cohort. **B, C** The number of core genes in the overlapping upregulated pathways between BCa group and normal group (**B**) and between RAB14 high expression group and low expression group (**C**) based on TCGA BLCA cohort. **D** The three sugar metabolism pathways activated concurrently among the overlapping upregulated pathways. **E** The intersect between our RAB14 chip data (knockdown RAB14 in 5637 cells) (blue) and core genes of the three sugar metabolism pathways (red and green). **F** Correlation analysis between RAB14 and PGM3 expression from TIMER database.

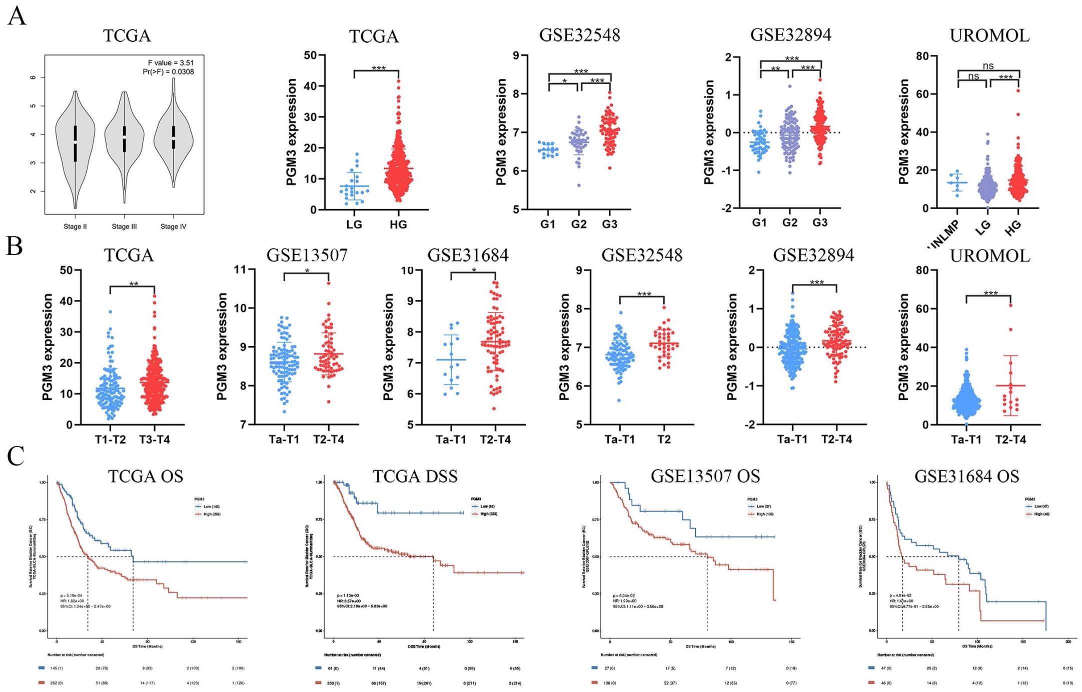

Figure S2. A The expression of PGM3 in various clinical stages or pathological grades of BCa from TCGA, GSE32548, GSE32894 and UROMOL cohort. **B** The expression of PGM3 in different T stages of BCa from TCGA, GSE13507, GSE31684, GSE32548, GSE32894 and UROMOL cohort. **C** The prognostic value of PGM3 in BCa from TCGA, GSE13507 and GSE31684 datasets. Data are presented as mean ± SD, the differences between groups were compared by student's t test, (*) represents *P*<0.05, (**) represents *P*<0.01, (***) represents *P*<0.001.

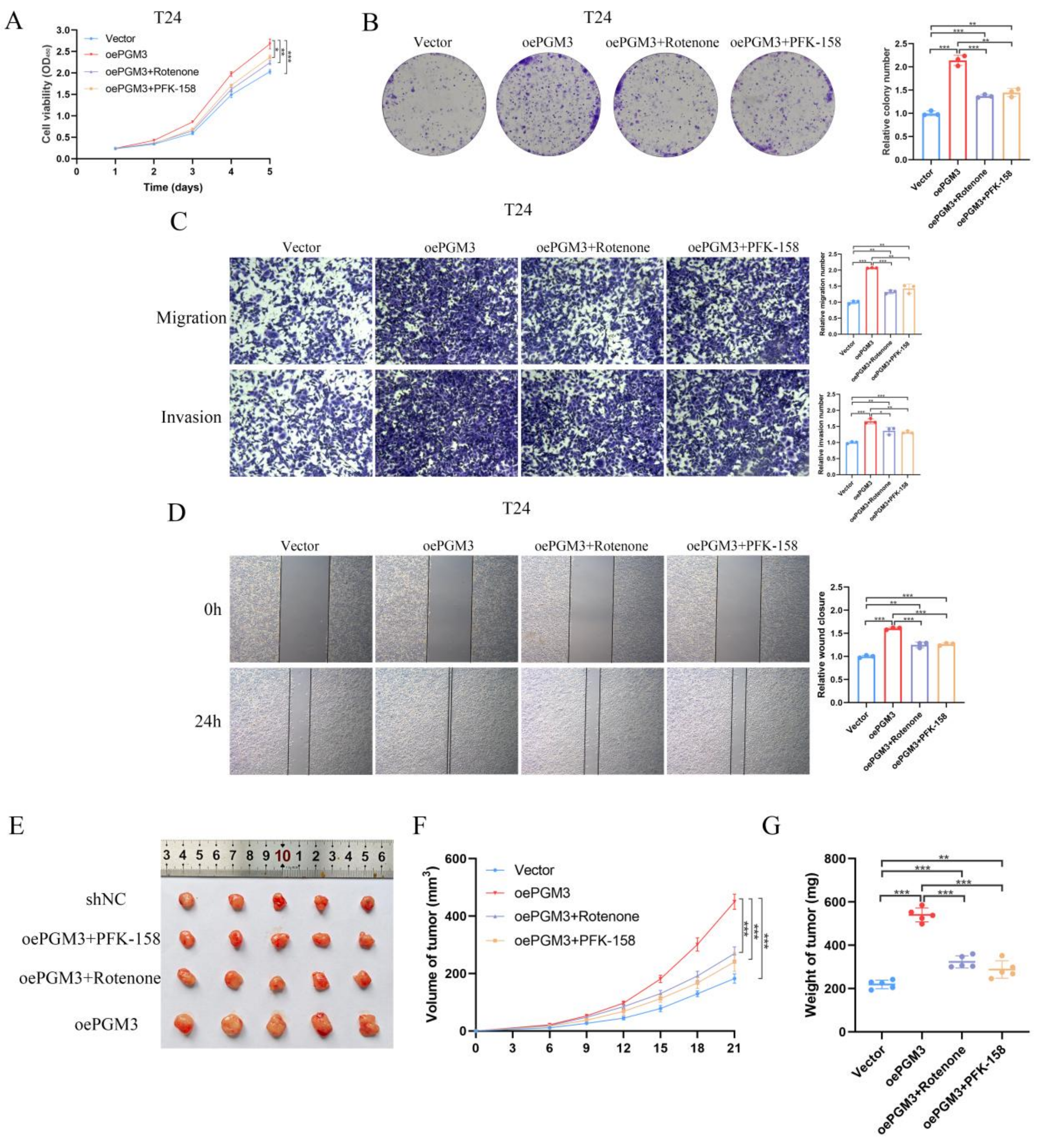

Figure S3. A, B CCK-8 (**A**) and colony formation (**B**) assays to determine the effect of PGM3 overexpression, and combined with OXPHOS inhibitor rotenone or glycolysis inhibitor PFK-158 treatment on cell proliferation in T24 cells. **C, D** Transwell (**C**) and wound healing (**D**) assays to determine the influence of PGM3 overexpression, and combined with OXPHOS inhibitor rotenone or glycolysis inhibitor PFK-158 treatment on migration and invasion ability in T24 cells. **E-F** The comparisons of tumor volume (**E**), tumor growth rate (**F**) and tumor weight (**G**) in BALB/c nude mice between control, PGM3 overexpression, PGM3 overexpression combined with OXPHOS inhibitor rotenone or glycolysis inhibitor PFK-158 treatment groups. Data are presented as mean ± SD, the differences between groups

were compared by student's t test, (*) represents *P*<0.05, (**) represents *P*<0.01, (***) represents *P*<0.001.

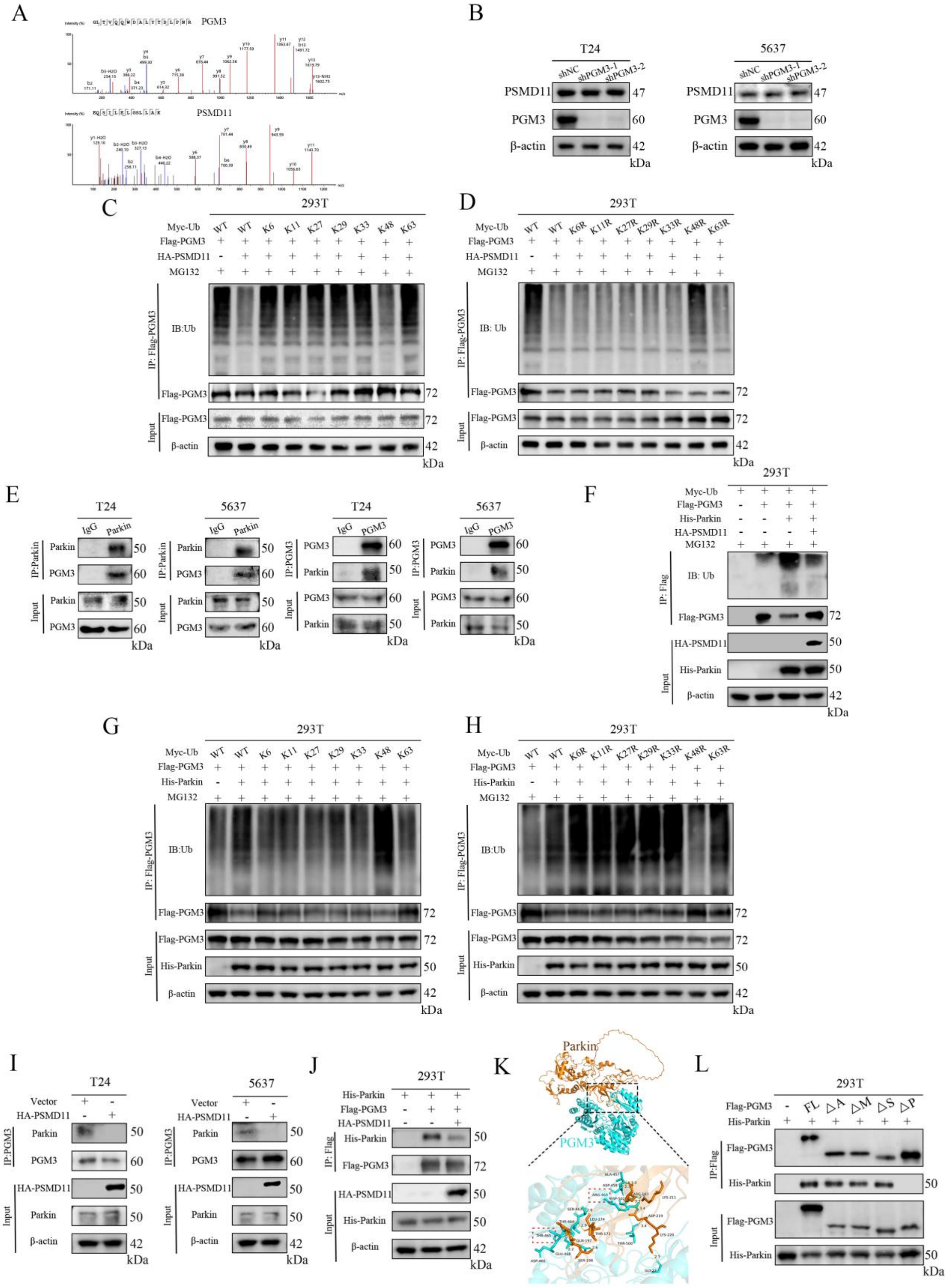

Figure S4. A Mass spectrogram of PGM3 and PSMD11 protein. **B** Western blot analysis to determine the protein level change of PSMD11 after PGM3 knockdown in

T24 and 5637 cells. **C, D** Co-IP and WB analysis to identify the specific polyubiquitination chain type of PGM3 affected by PSMD11. **E** Validation of interaction between endogenous PGM3 and Parkin in T24 and 5637 cells. **F** The effect of PSMD11 overexpression on Parkin-mediated ubiquitination of PGM3 in 293T cells **G, H** Co-IP and WB analysis to identify the specific polyubiquitination chain type of PGM3 affected by Parkin. **I, J** The effect of PSMD11 overexpression on the endogenous (**I**) and exogenous (**J**) interaction between Parkin and PGM3. **K** The molecular docking model of Parkin and PGM3. **L** Co-IP and WB performed the interaction analysis between Parkin and full length and domain-deletion mutant PGM3 in 293T cells.

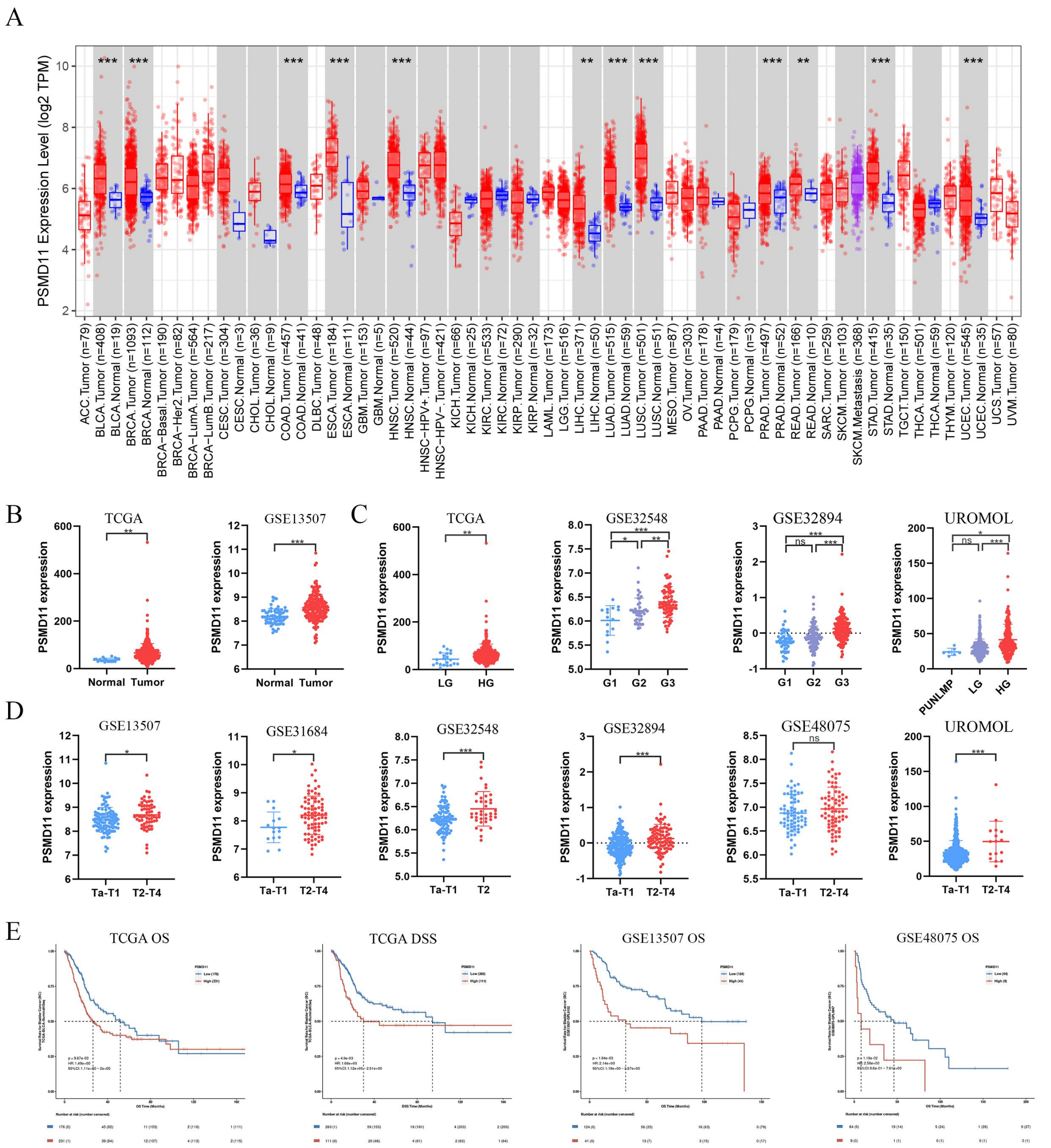

Figure S5. A The differential expression of PSMD11 between tumor and normal tissues in Pan-cancer from TIMER database. **B** The expression of PSMD11 between BCa and normal tissues from TCGA BLCA and GSE13507 cohort. **C** The expression of PSMD11 in various clinical stages or pathological grades of BCa from TCGA, GSE32548, GSE32894 and UROMOL cohort. **D** The expression of PSMD11 in different T stages of BCa from GSE13507, GSE31684, GSE32548, GSE32894, GSE48075 and UROMOL cohort. **E** The prognostic value of PSMD11 in BCa from TCGA, GSE13507 and GSE48075 datasets. Data are presented as mean ± SD, the

differences between groups were compared by student's t test, (*) represents *P*<0.05, (**) represents *P*<0.01, (***) represents *P*<0.001.

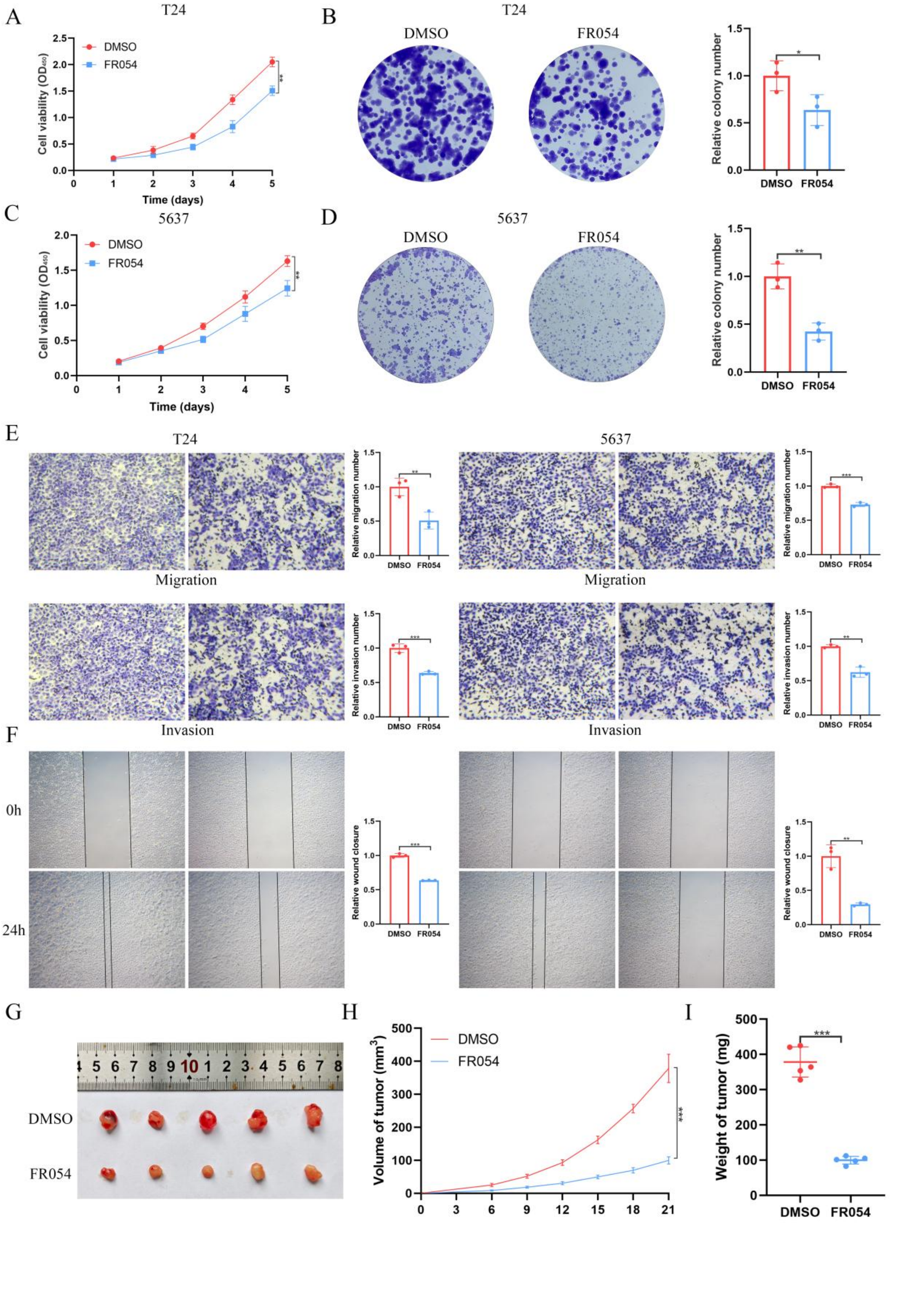

Figure S6. A-D CCK-8 (**A, C**) and colony formation (**B, D**) assays to determine the effect of the treatment with PGM3 inhibitor FR054 (0.5 mM) for 24h on cell

proliferation in T24 and 5637 cells. **E, F** Transwell (**E**) and wound healing (**F**) assays to determine the influence of the treatment with FR054 (0.5 mM) for 24h on migration and invasion ability in T24 and 5637 cells. **G-I** The comparisons of tumor volume (**G**), tumor growth rate (**H**) and tumor weight (**I**) in BALB/c nude mice administered with DMSO or FR054 (500 mg/kg/day, intraperitoneal injection after six days of T24 cells inoculation). Data are presented as mean ± SD, the differences between groups were compared by student's t test, (*) represents $P<0.05$, (**) represents $P<0.01$, (***) represents $P<0.001$.
