# Supplementary material for: PSMD11 stabilizes PGM3 by antagonizing Parkin to promote bladder cancer progression through energy metabolism reprogramming
Source: Cell Death Dis. 2026 Apr 6;17(1):457. doi: 10.1038/s41419-026-08691-4 (PMC13184242; doi:10.1038/s41419-026-08691-4)

Figure 1F

β-actin 1-5

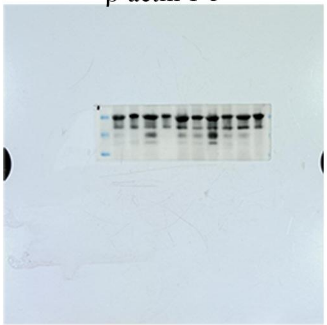

β-actin6-10

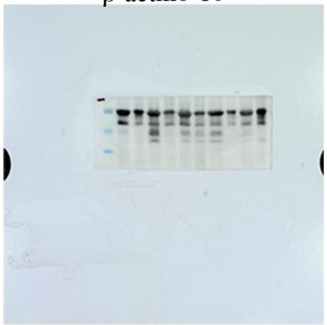

PGM3 1-5

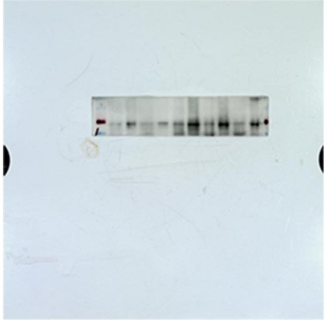

PGM3 6-10

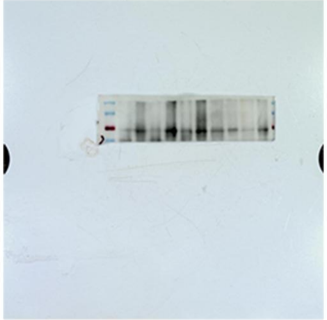

Figure 2A

β-actin

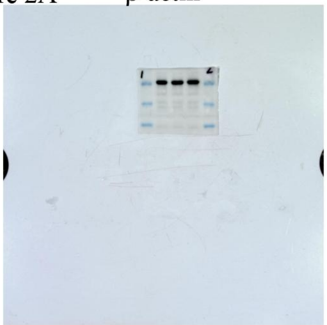

PGM3

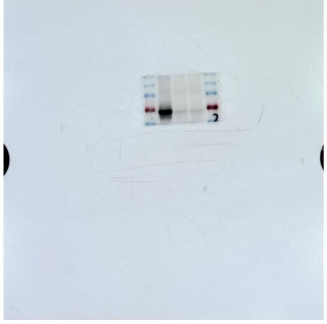

Figure 2D

β-actin

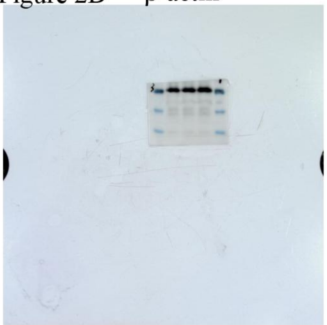

PGM3

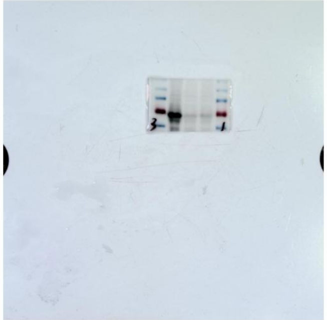

Figure 3U

T24

β-actin

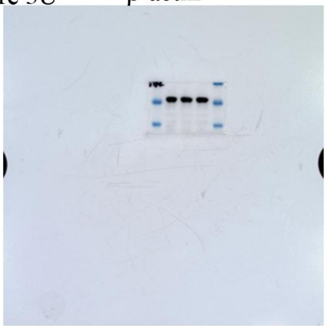

PGM3

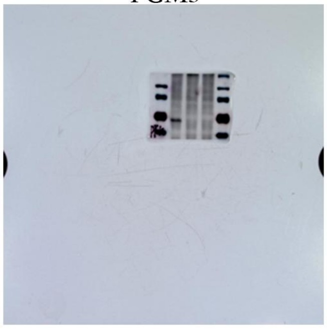

PFKFB3

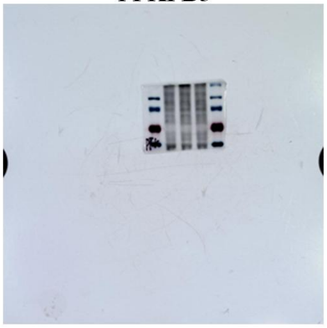

ENO1

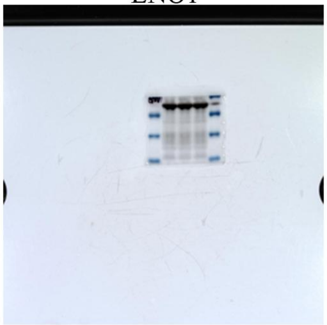

HK2

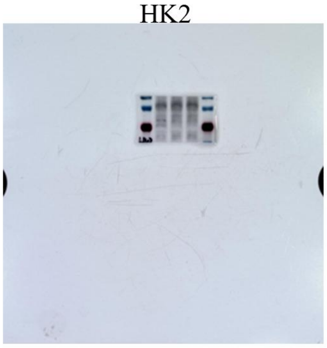

ND 5

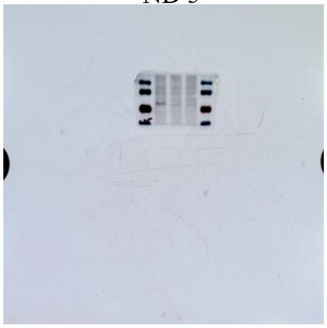

CYTB

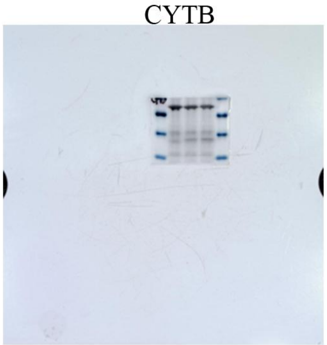

5637

β-actin

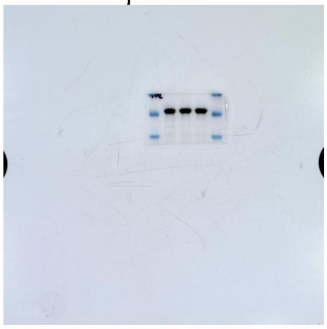

PGM3

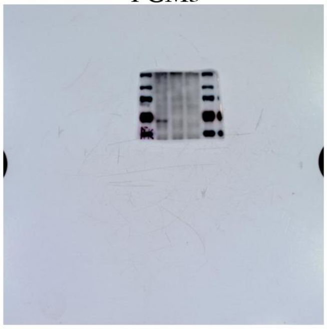

PFKFB3

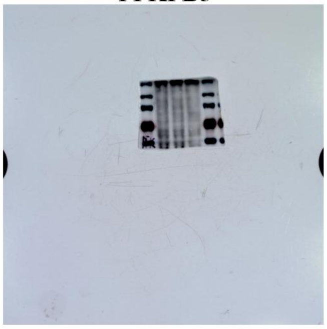

ENO1

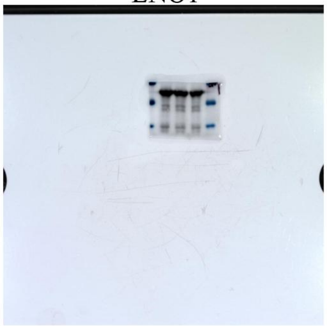

HK2

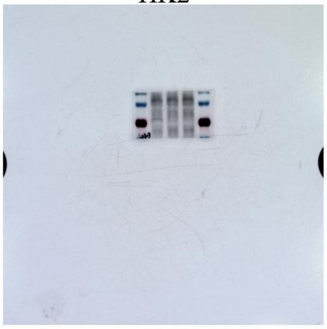

ND 5

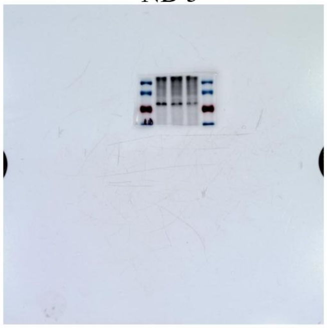

CYTB

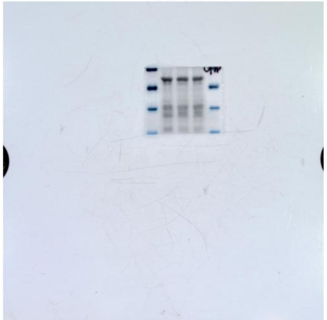

Figure 4E

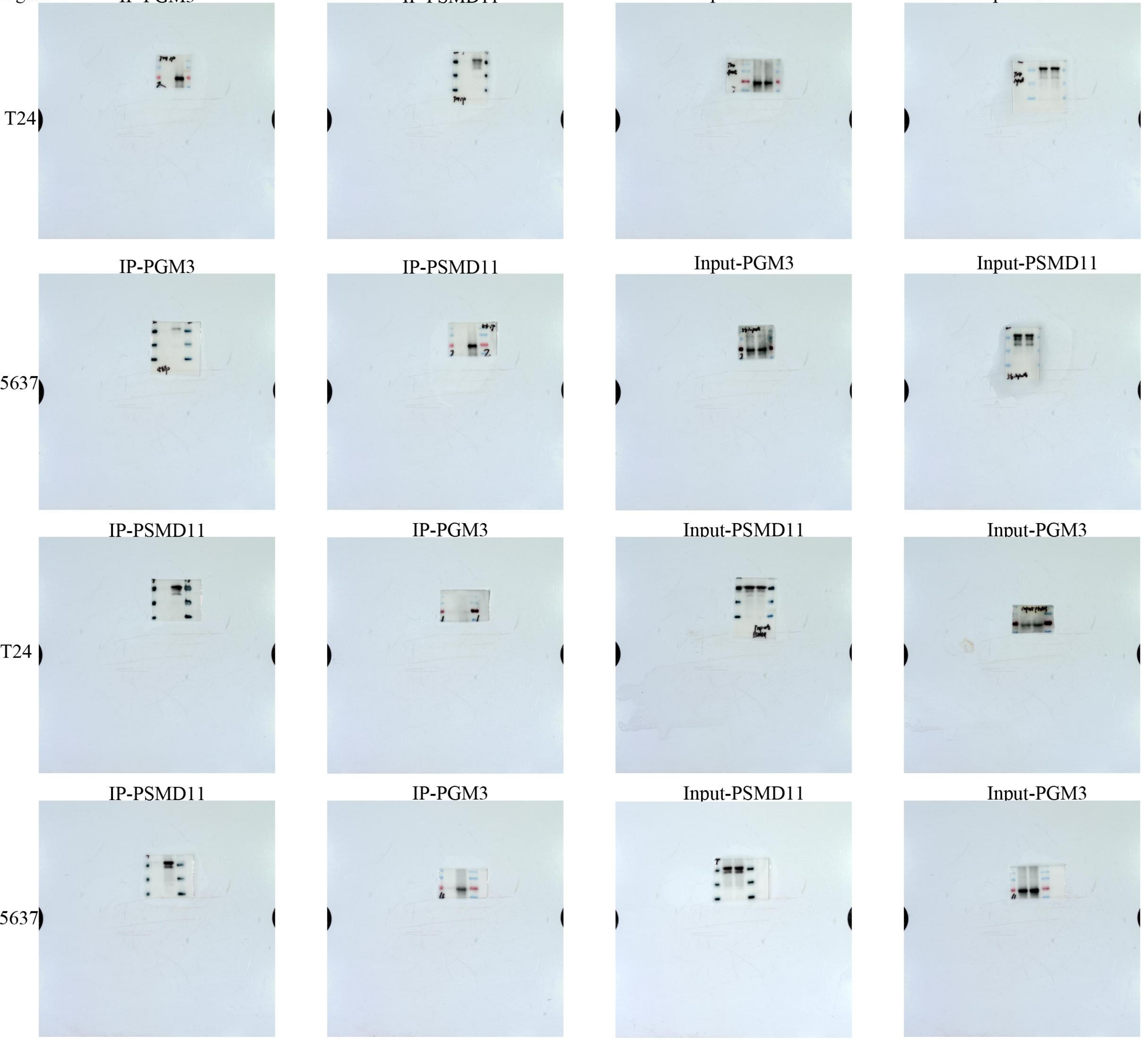

Figure 4F

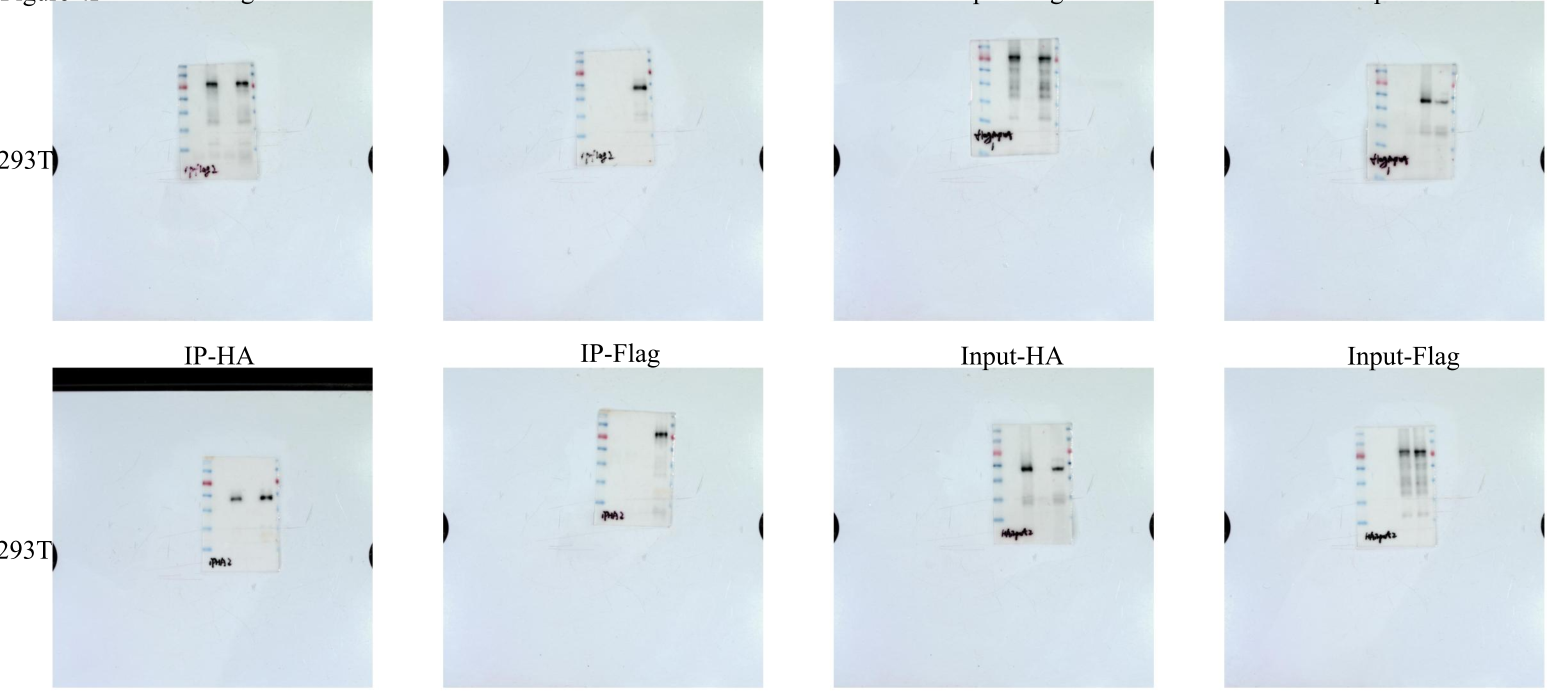

Figure 4I

T24 β-actin | PSMD11 | PGM3 | 5637 β-actin | PSMD11 | PGM3

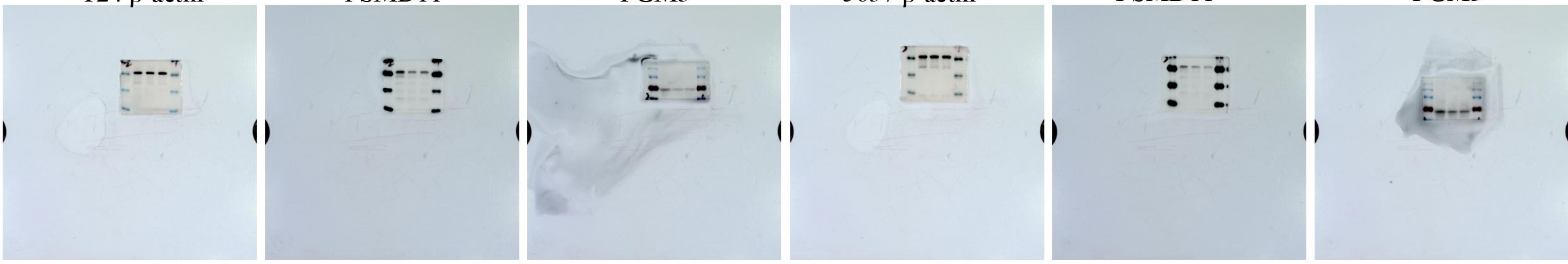

Figure 4J

T24 β-actin | PSMD11 | PGM3

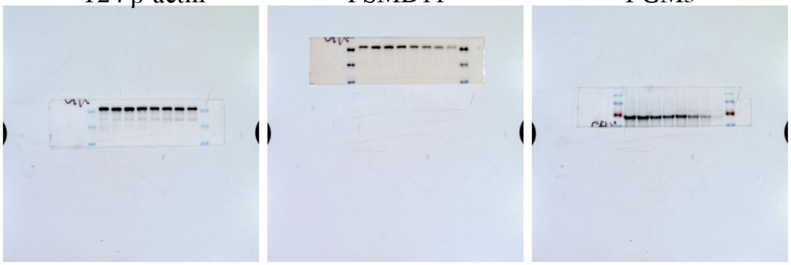

Figure 4K

5637 β-actin | PSMD11 | PGM3

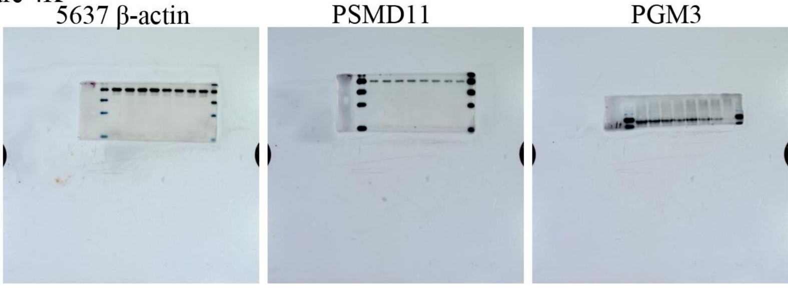

Figure 4L

T24 β-actin | PSMD11 | PGM3 | 5637 β-actin | PSMD11 | PGM3

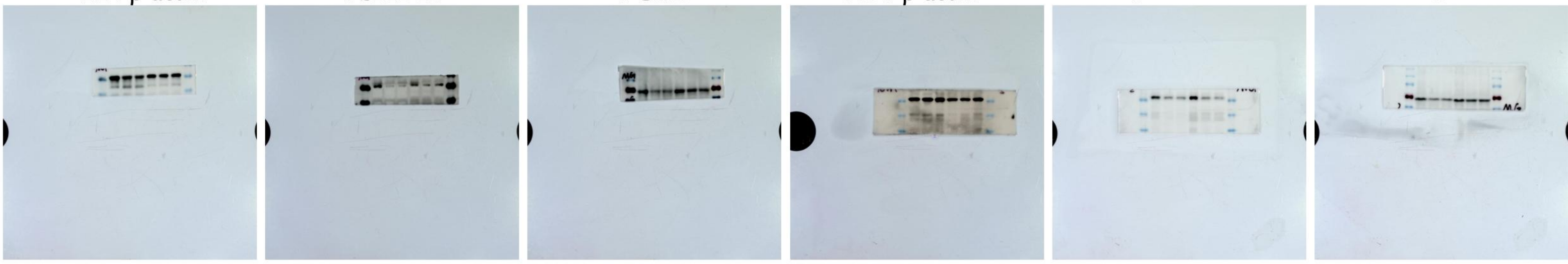

Figure 4M

T24 β-actin | PSMD11 | PGM3 | 5637 β-actin | PSMD11 | PGM3

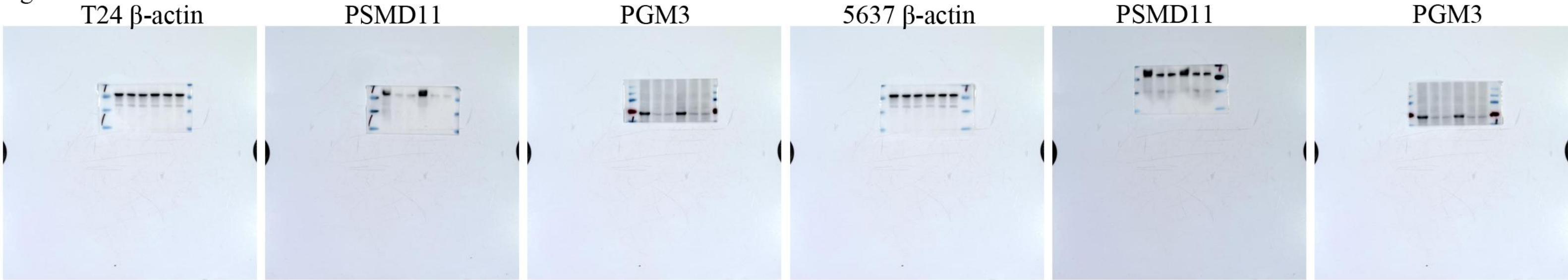

Figure 4N

T24 inpu β-actin | input PSMD11 | IP PGM3 | IP Ub

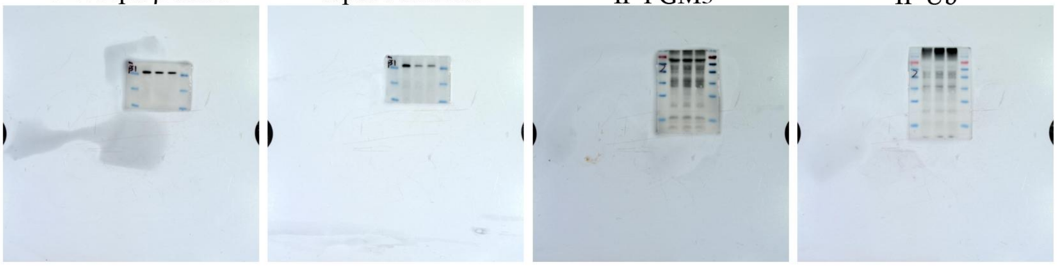

5637 input β-actin | input PSMD11 | IP PGM3 | IP Ub

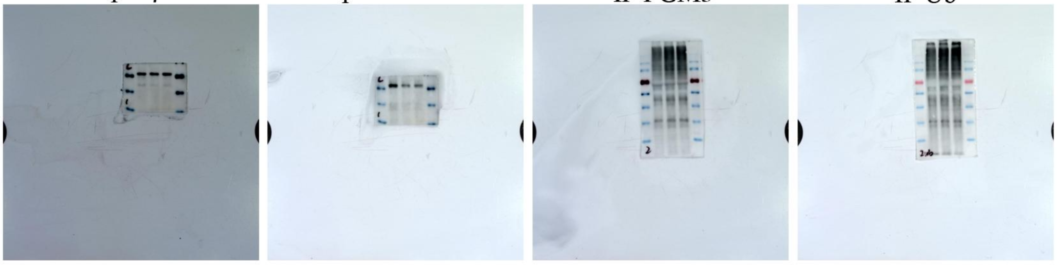

Figure 4O

input β-actin | input Flag | IP Flag | IP Ub

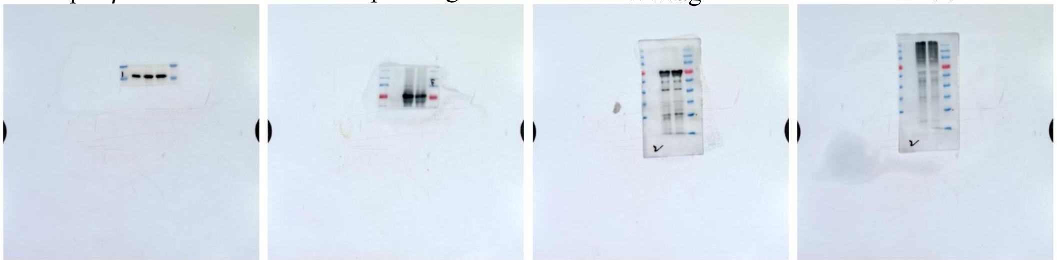

Figure 4P

input β-actin | input HA | input Flag | IP Flag | IP Ub

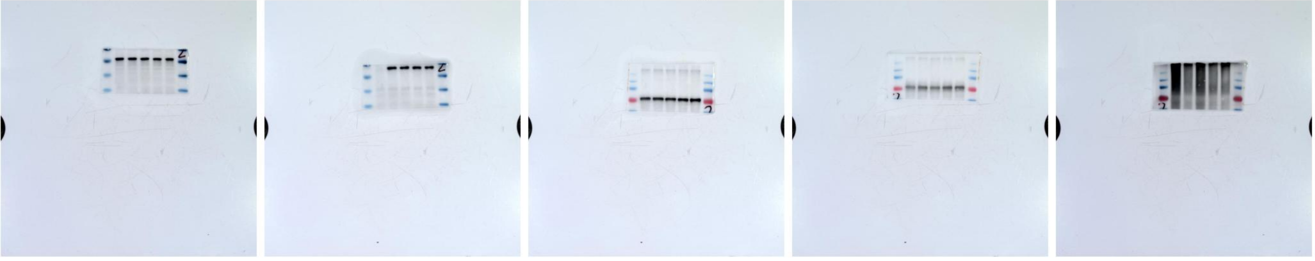

Figure 4Q

T24 β-actin | HA-PSMD11 | Flag-PGM3 | 5637 β-actin | HA-PSMD11 | Flag-PGM3

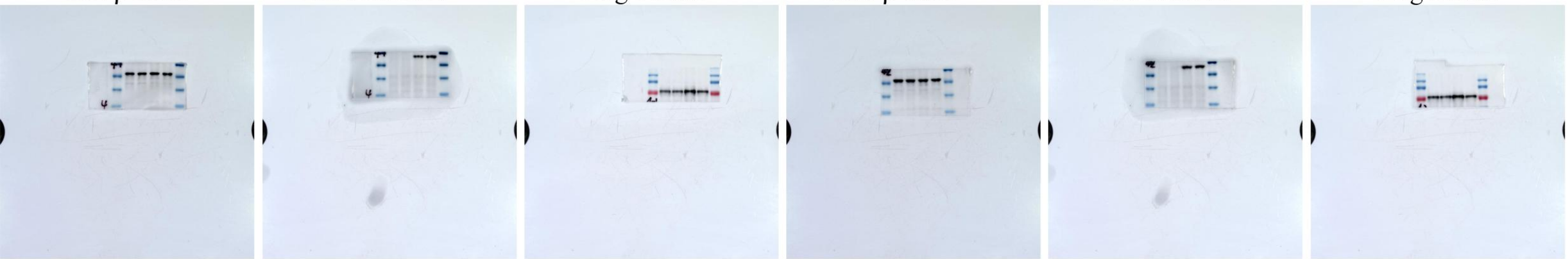

Figure 5B

input HA | input Flag | IP HA | IP Flag

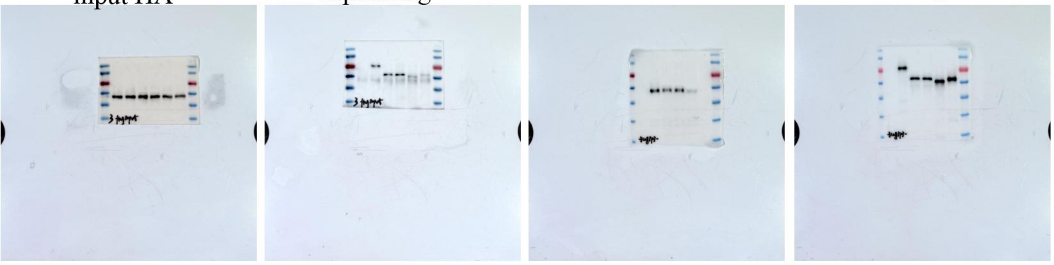

Figure 5D

input β-actin | input HA | input Flag | IP HA | IP Flag

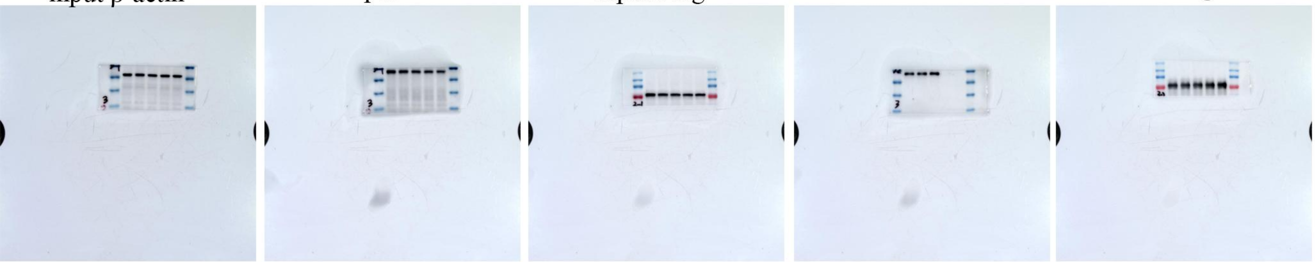

Figure 5E

T24 β-actin | HA-PSMD11 | Flag-PGM3 | 5637 β-actin | HA-PSMD11 | Flag-PGM3

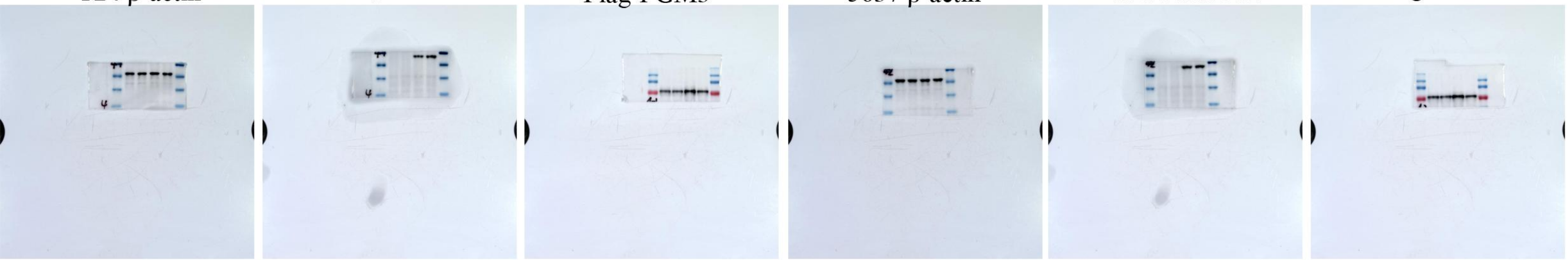

Figure 5F

input Flag | input His | IP Flag | IP His

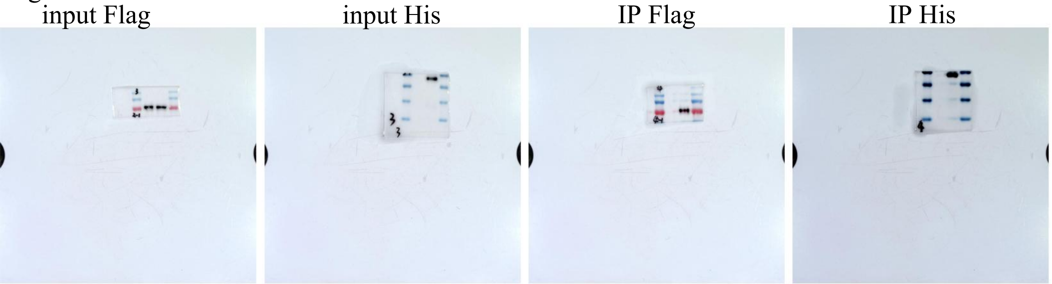

input His | input Flag | IP His | IP Flag

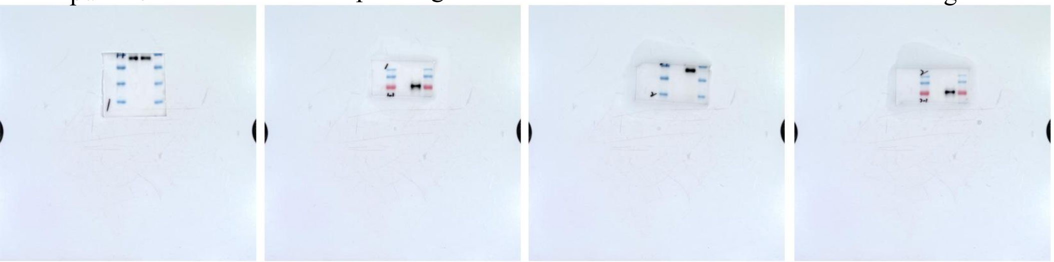

Figure 5I

T24 β-actin His-Parkin PGM3 5637 β-actin His-Parkin PGM3

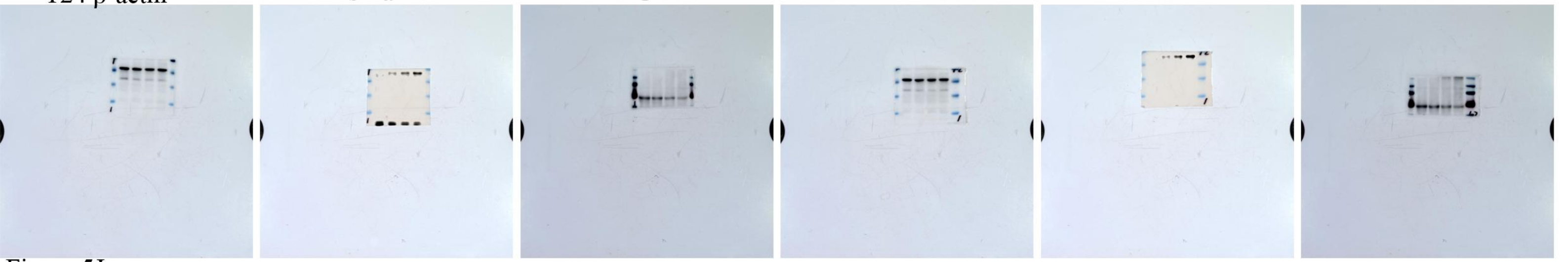

Figure 5J

β-actin His-Parkin PGM3

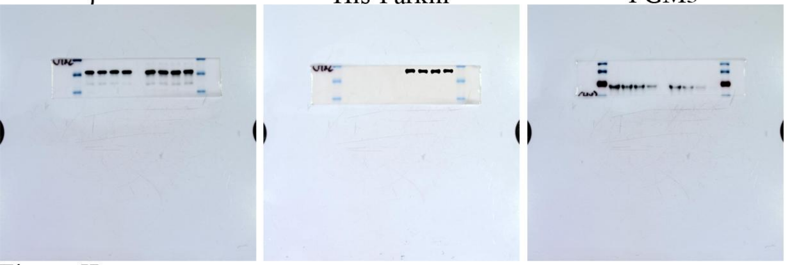

Figure 5K

β-actin His-Parkin PGM3

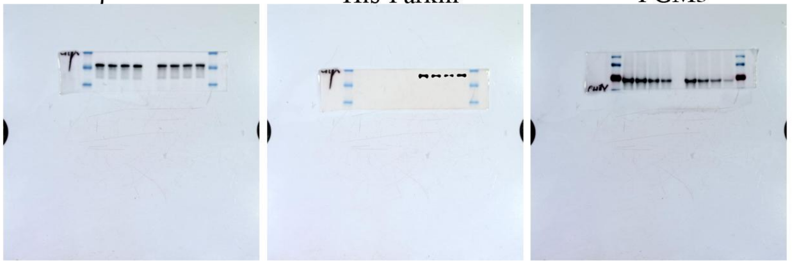

Figure 5L

T24 β-actin His-Parkin PGM3 5637 β-actin His-Parkin PGM3

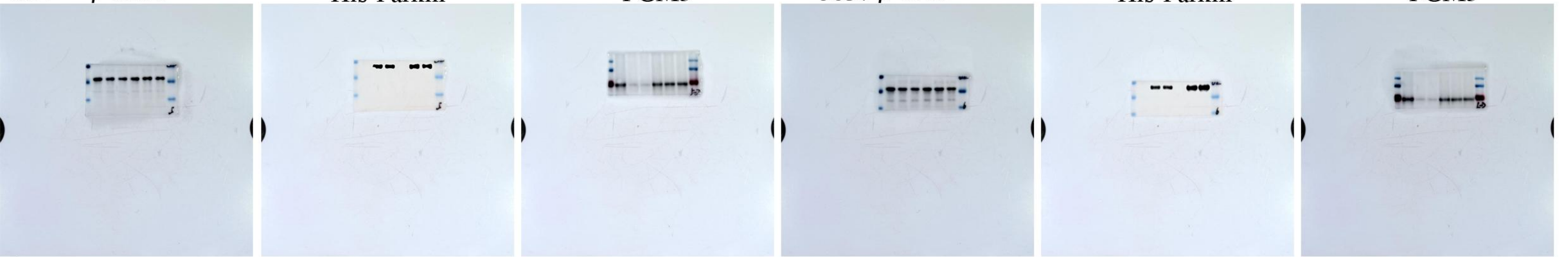

Figure 5M

T24 β-actin His-Parkin PGM3 5637 β-actin His-Parkin PGM3

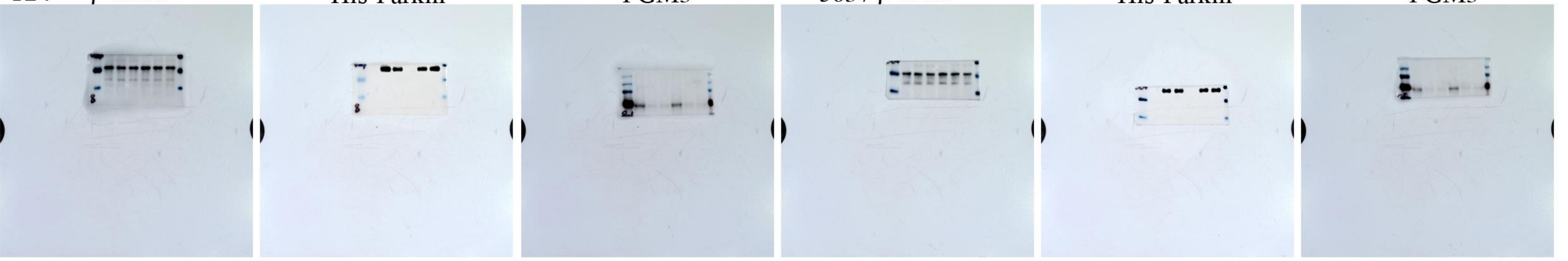

Figure 5N

T24 input β-actin input Parkin IP PGM3 IP Ub

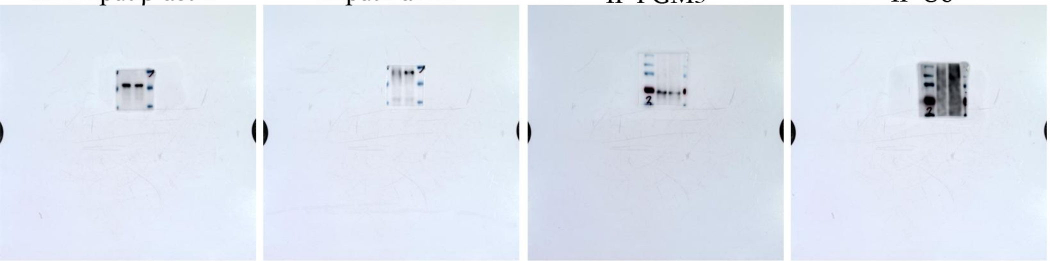

5637 input β-actin input Parkin IP PGM3 IP Ub

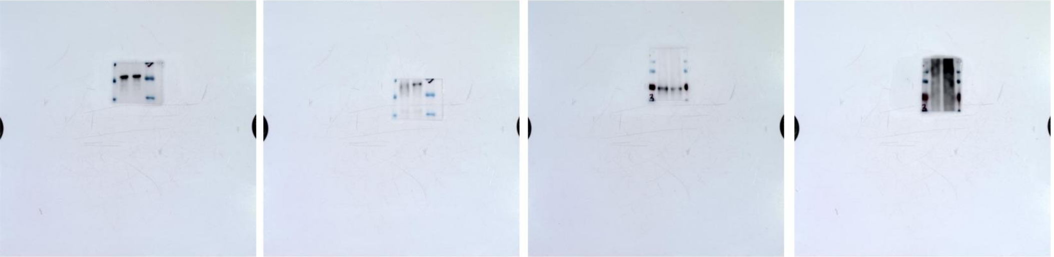

Figure 5O

input β-actin input His input Flag IP Flag IP Ub

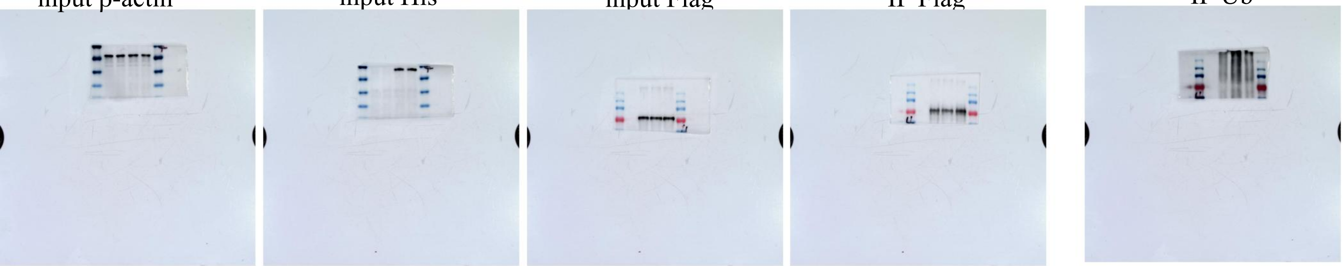

Figure 5P

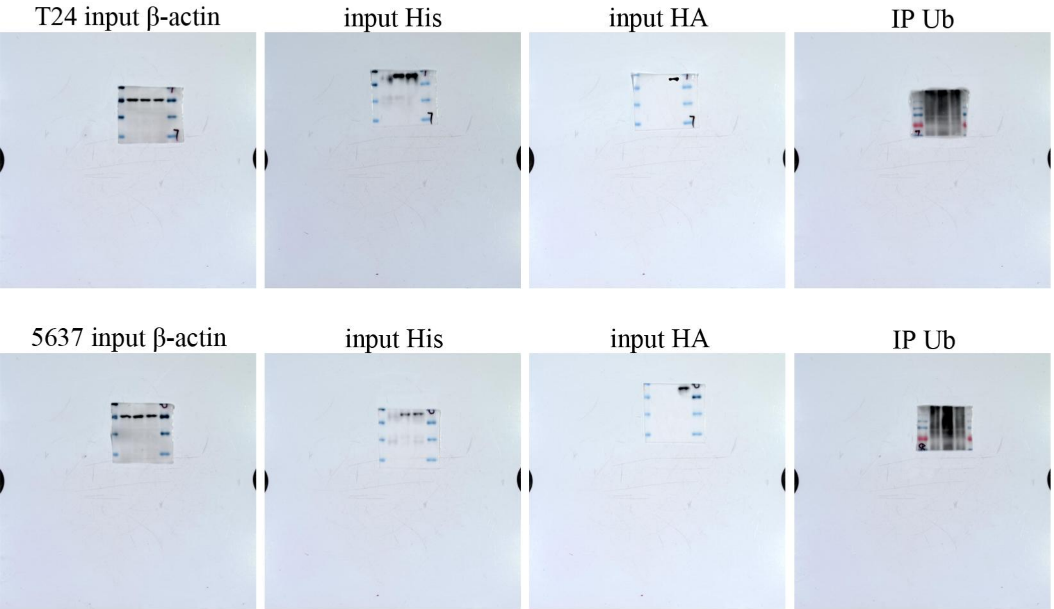

Figure 6G

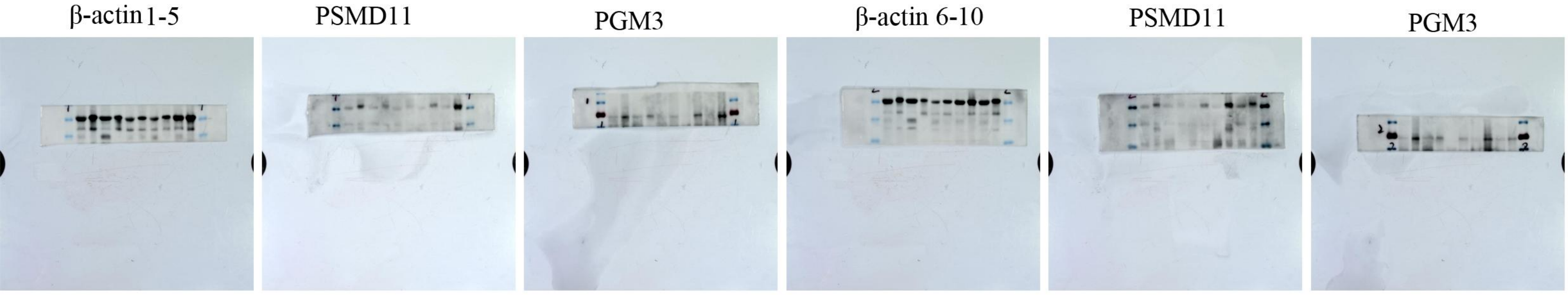

Figure 7A

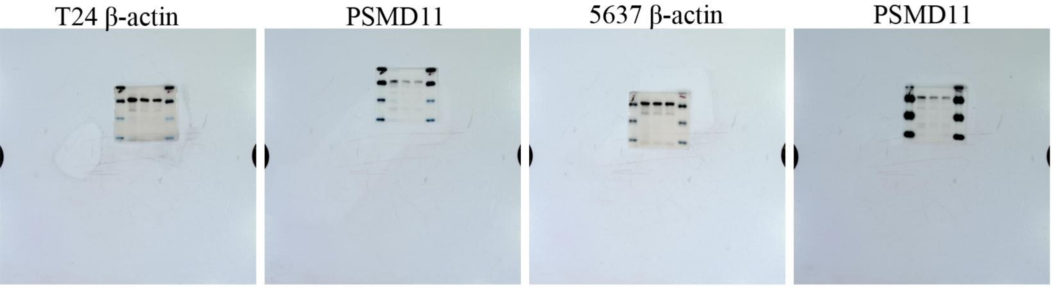

Figure S4B

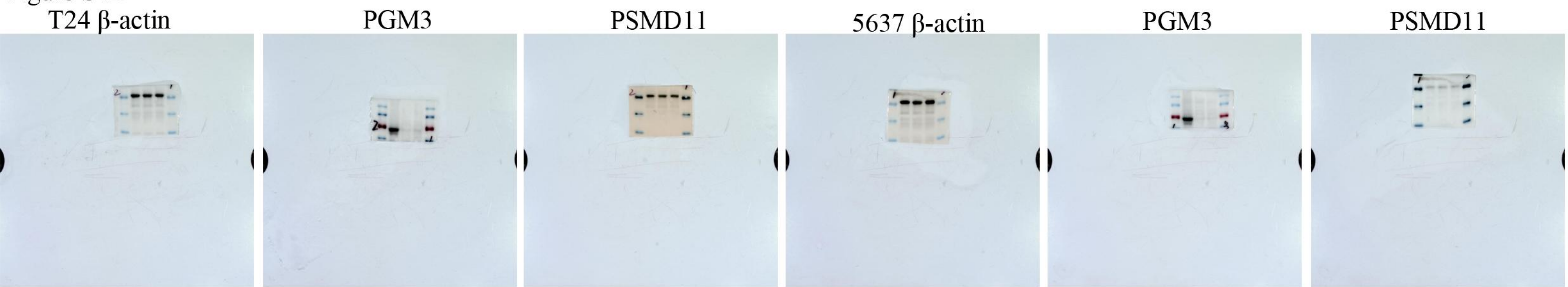

Figure S4C

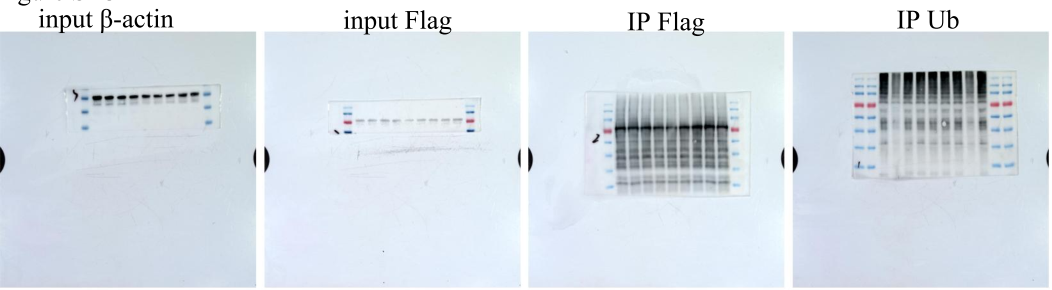

Figure S4D

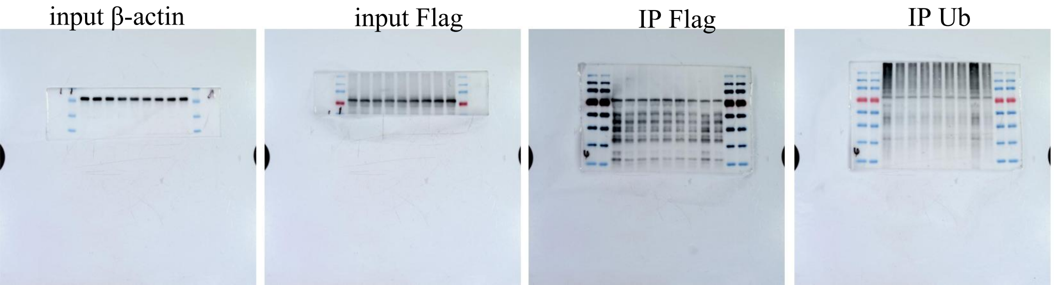

Figure S4E

T24 input PGM3 | input Parkin | IP PGM3 | IP Parkin

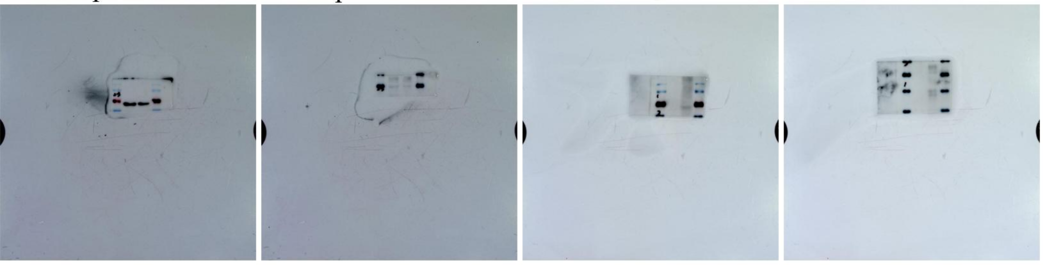

5637 input PGM3 | input Parkin | IP PGM3 | IP Parkin

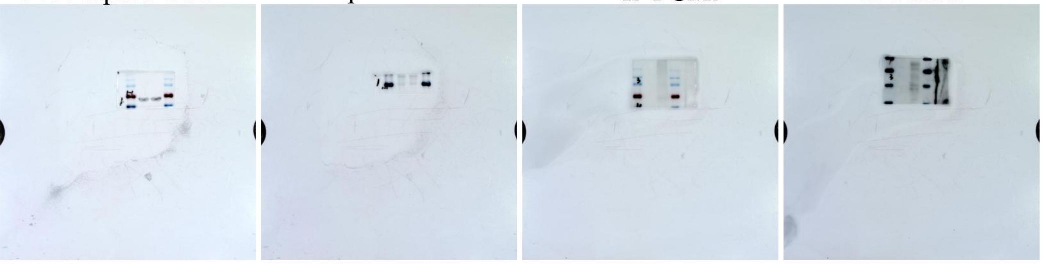

T24 input Parkin | input PGM3 | IP Parkin | IP PGM3

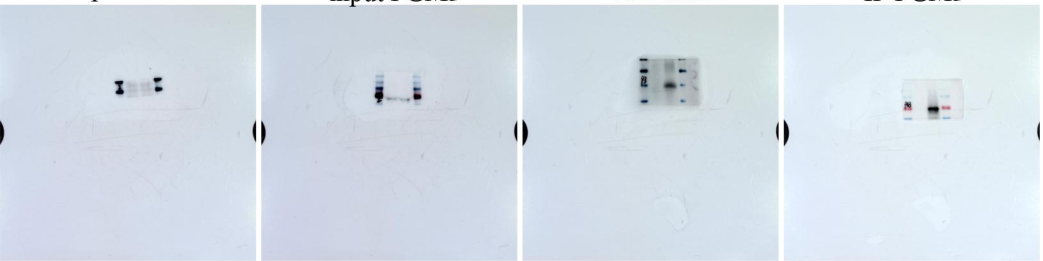

5637 input Parkin | input PGM3 | IP Parkin | IP PGM3

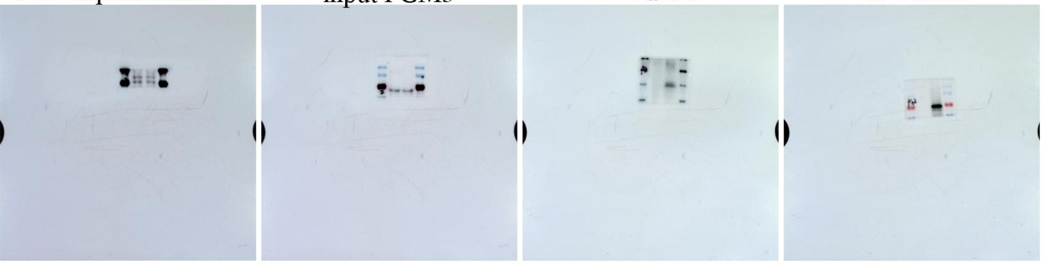

Figure S4F

input β-actin | input His | input HA | IP Flag | IP Ub

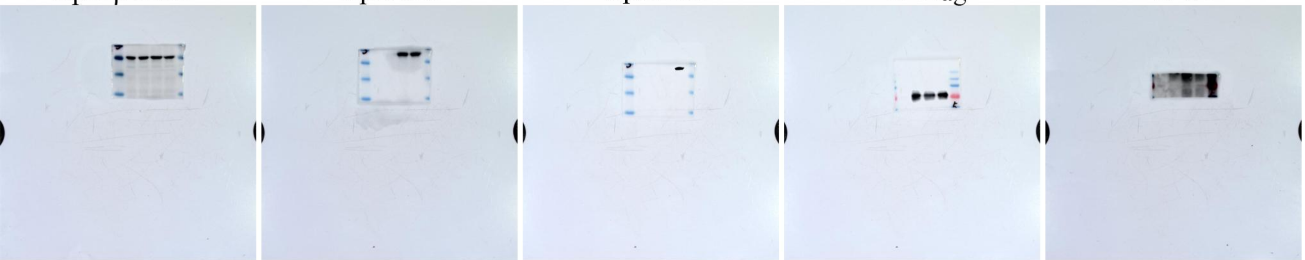

Figure S4G

input β-actin | input His | input Flag | IP Flag | IP Ub

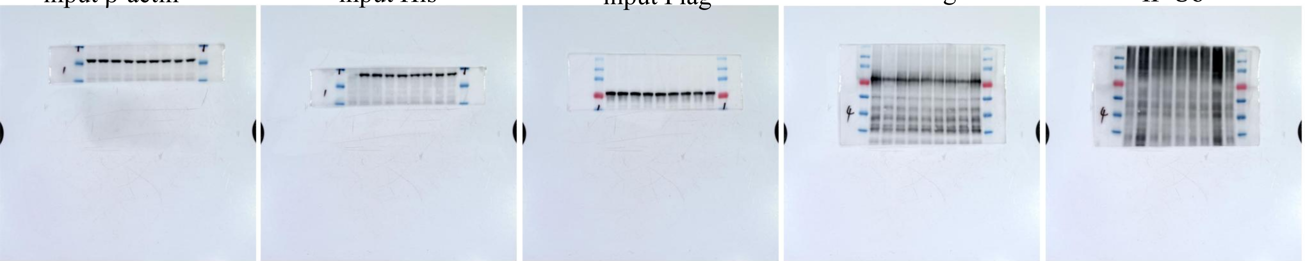

Figure S4H

input β-actin | input His | input Flag | IP Flag | IP Ub

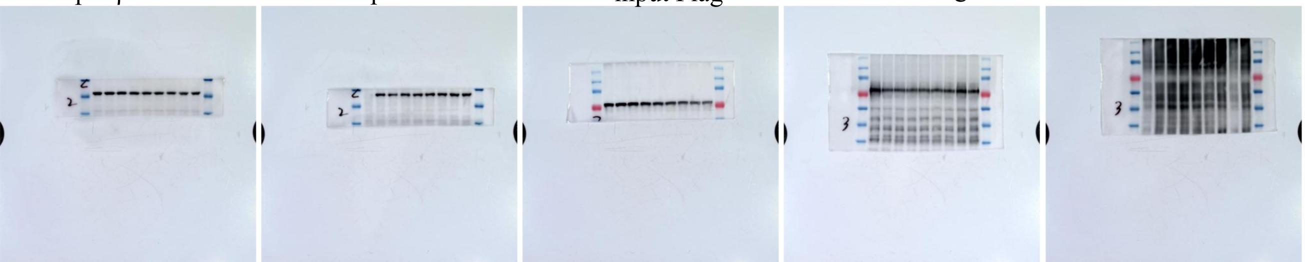

Figure S4I

T24 input β-actin | input Parkin | input HA | IP PGM3 | IP Parkin

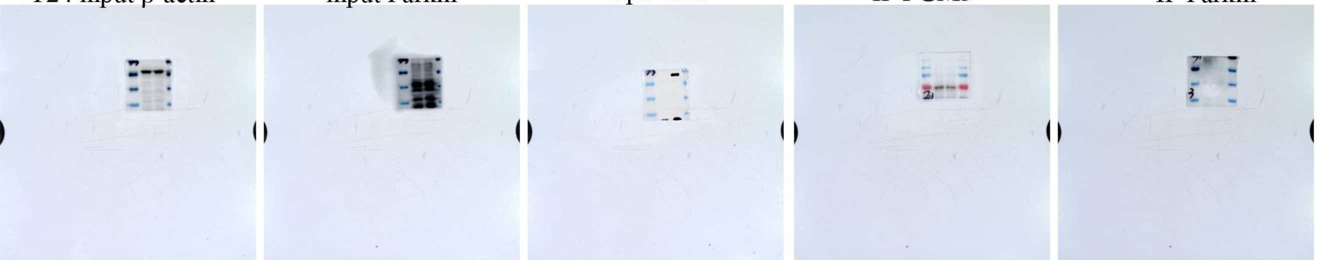

5637 input β-actin | input Parkin | input HA | IP PGM3 | IP Parkin

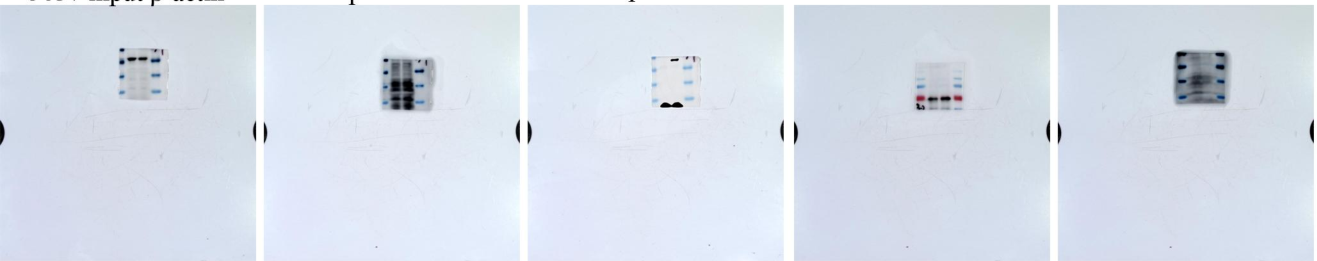

Figure S4J

input β-actin | input His | input HA | IP Flag | IP His

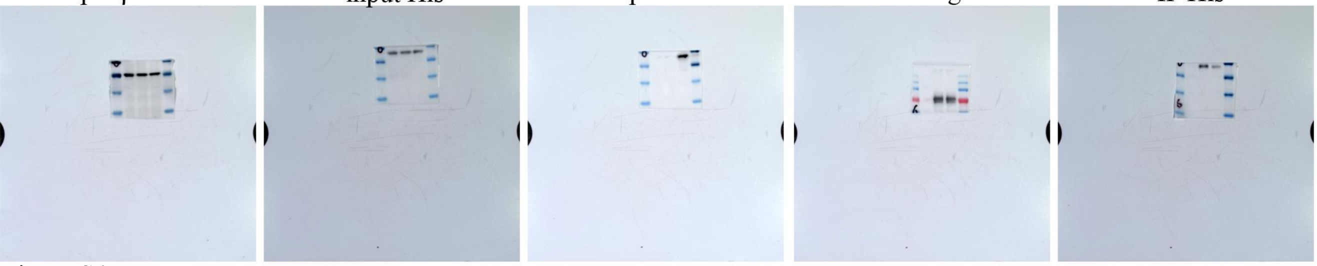

Figure S4L

input His | input Flag | IP His | IP Flag

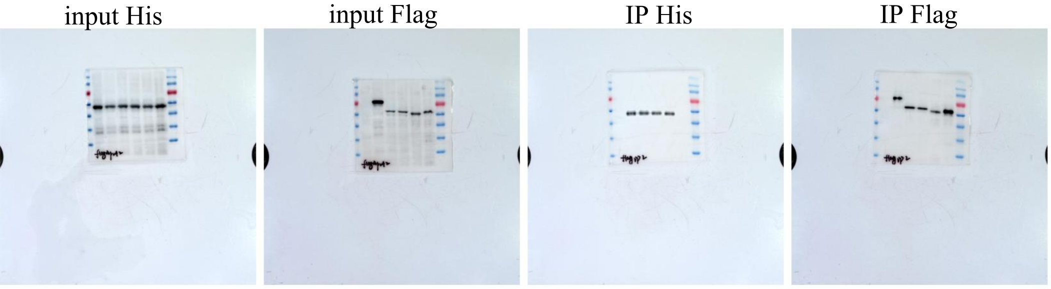

Supplement: Supplementary file 3 — uncropped gels and blots images [file 41419_2026_8691_MOESM3_ESM.pdf]
